# ZfpA-regulated chitin synthesis in *Aspergillus fumigatus* hyphae determines fungicidal tip lysis by FksA-targeting antifungals

**DOI:** 10.1128/aac.01769-25

**Published:** 2026-06-04

**Authors:** Dante G. Calise, Madeline L. Michaelis, Sung Chul Park, Andrew S. Wagner, Nancy P. Keller

**Affiliations:** 1 Department of Medical Microbiology and Immunology, University of Wisconsin–Madison 732057, Madison, Wisconsin, USA; 2 Department of Genetics, University of Wisconsin–Madison 312671, Madison, Wisconsin, USA; 3 Department of Biological Sciences, Bowling Green State University 110004, Bowling Green, Ohio, USA; 4 Department of Plant Pathology, University of Wisconsin–Madison 312673, Madison, Wisconsin, USA; University of Iowa, Iowa City, Iowa, USA

**Keywords:** chitin synthase, caspofungin, antifungals, *Aspergillus fumigatus*, fungal genetics, echinocandin

## Abstract

*Aspergillus fumigatus* poses a serious threat to the health of a growing immunocompromised population. Invasive aspergillosis infections have one of the highest mortality rates of any disease, and treatment options are extremely limited. The β-1,3-glucan synthase-inhibiting echinocandins are one of only three classes of drugs approved to treat these infections. However, their clinical efficacy against *Aspergillus* is considered fungistatic. Disruption of proper cell wall synthesis at the growing tip of hyphae can result in the lysis of the apical tip compartment. The oxylipin-associated transcription factor ZfpA is known to regulate a growth program in response to echinocandins that blocks the tip lytic action of these drugs. It is known that ZfpA positively regulates hyphal cell wall chitin, which is also found to be upregulated in response to echinocandins. Here, we find that ZfpA is activated broadly by cell wall stress but confers protection against only β-1,3-glucan targeting antifungals including the lipopeptide echinocandins and the novel terpene fungerp enfumafungin. In addition, we show that at least four of the eight chitin synthases in *A. fumigatus* are important for echinocandin tolerance, contributing to a multifactorial response to tolerate β-1,3-glucan depletion downstream of the transcription factor ZfpA. We find that the enzymes ChsF and ChsG, specifically, play opposing roles in resistance to apical tip lysis due to inhibition of β-1,3-glucan synthase.

## INTRODUCTION

*Aspergillus fumigatus* is an important pathogen of many animals and is found globally as an environmental saprophyte. Humans are estimated to inhale hundreds of *A. fumigatus* conidia every day, but a healthy immune response effectively kills and clears these asexual spores from the lung, preventing hyphal growth. However, in highly immunocompromised individuals, successful germination of inhaled conidia and tissue-invasive hyphal growth causes a serious infection known as invasive aspergillosis (IA) (1). Relative to bacterial pathogens, there are few known fungal-specific molecular processes that can be targeted by antimicrobial drugs, largely due to significant functional conservation between fungal and animal biology. As a result, primary treatment of invasive fungal infections is currently limited to only three approved classes of antifungal drugs—polyenes, azoles, and echinocandins—and no singular class is consistently effective against all fungal pathogens. Amphotericin B is the only member of the polyene class used for systemic infection and is generally effective at killing most fungal species. However, it can be highly cytotoxic in humans. It functions by binding ergosterol to disrupt the fungal cell membrane, but it can have a similar effect via analogous cholesterol binding in the plasma membrane of animal cells. In the case of IA, the azoles are considered the first-line therapy. These drugs also target the fungal cell membrane but do so instead by inhibition of ergosterol synthesis, thus reducing their human cytotoxicity. They are generally fungicidal against *A. fumigatus,* although increasing numbers of azole-resistant isolates are being reported, likely due to widespread use of these compounds as crop fungicides. In cases of azole treatment failure, salvage therapy is necessary with either amphotericin B or the echinocandin antifungals (2).

The echinocandins differ from the other two antifungal classes, in that they target the fungal cell wall rather than the cell membrane. This is an ideal drug target because animal cells lack a cell wall, so the risk of related cytotoxicity is lower. Specifically, these drugs non-competitively inhibit the enzyme FksA responsible for the synthesis of β-1,3-glucan, an important structural component of the cell wall (3). However, the composition of the fungal cell wall is not well conserved across species, resulting in varying efficacy of the echinocandins. While the echinocandins are generally highly fungicidal against the Saccharomycotina, such as pathogenic *Candida* spp., their activity against Pezizomycetes, including aspergilli, is more complex (4). These are relatively large molecules, which must be delivered intravenously, presenting an additional limitation in their clinical use. Recently, a novel antifungal triterpenoid drug, ibrexafungerp, has been approved for the treatment of vulvovaginal candidiasis and is in late-stage clinical trials for use in invasive fungal infections. Although structurally dissimilar to the lipopeptide derived echinocandins, this terpene-derived drug is also a potent inhibitor of β-1,3-glucan synthase, and its much smaller size allows for effective oral administration. It has been shown to have similar effects to the echinocandins on the growth of several fungi, including *A. fumigatus* (5). This inhibition is characterized by both a fungicidal lysis of the apical compartment at the growing tip and a fungistatic stunting of hyphal growth (6).

We previously showed that production of the endogenous *Aspergillus* oxylipin (5*S*,8*R*,9*Z*,12*Z*)-5,8-dihydroxy-9,12-octadecadienoic acid (5,8-diHODE) by the dioxygenase PpoA is induced by echinocandins via the transcription factor ZfpA. ZfpA and PpoA cooperate in a positive feedback loop to protect specifically against the fungicidal tip lysis caused by echinocandins. Furthermore, we found that 5,8-diHODE and the echinocandin caspofungin (CSF) both increase cell wall chitin in an additive manner, which we hypothesized may structurally compensate for a lack of β-1,3-glucan (7). Here, we demonstrate a conserved role for the PpoA/ZfpA axis in protection from enfumafungin (ENF), the ibrexafungerp precursor. We further assess individual mutants of all eight chitin synthases in the *A. fumigatus* strain Af293 (8) and find that four of these proteins contribute to the tolerance of β-1,3-glucan synthase-targeting antifungals. However, regulation of hyphal chitin by the oxylipin-responsive transcription factor ZfpA does not fully explain its role in the tolerance of these antifungals.

## RESULTS

### ZfpA and PpoA form a positive feedback loop in response to echinocandins

**Fig 1:**
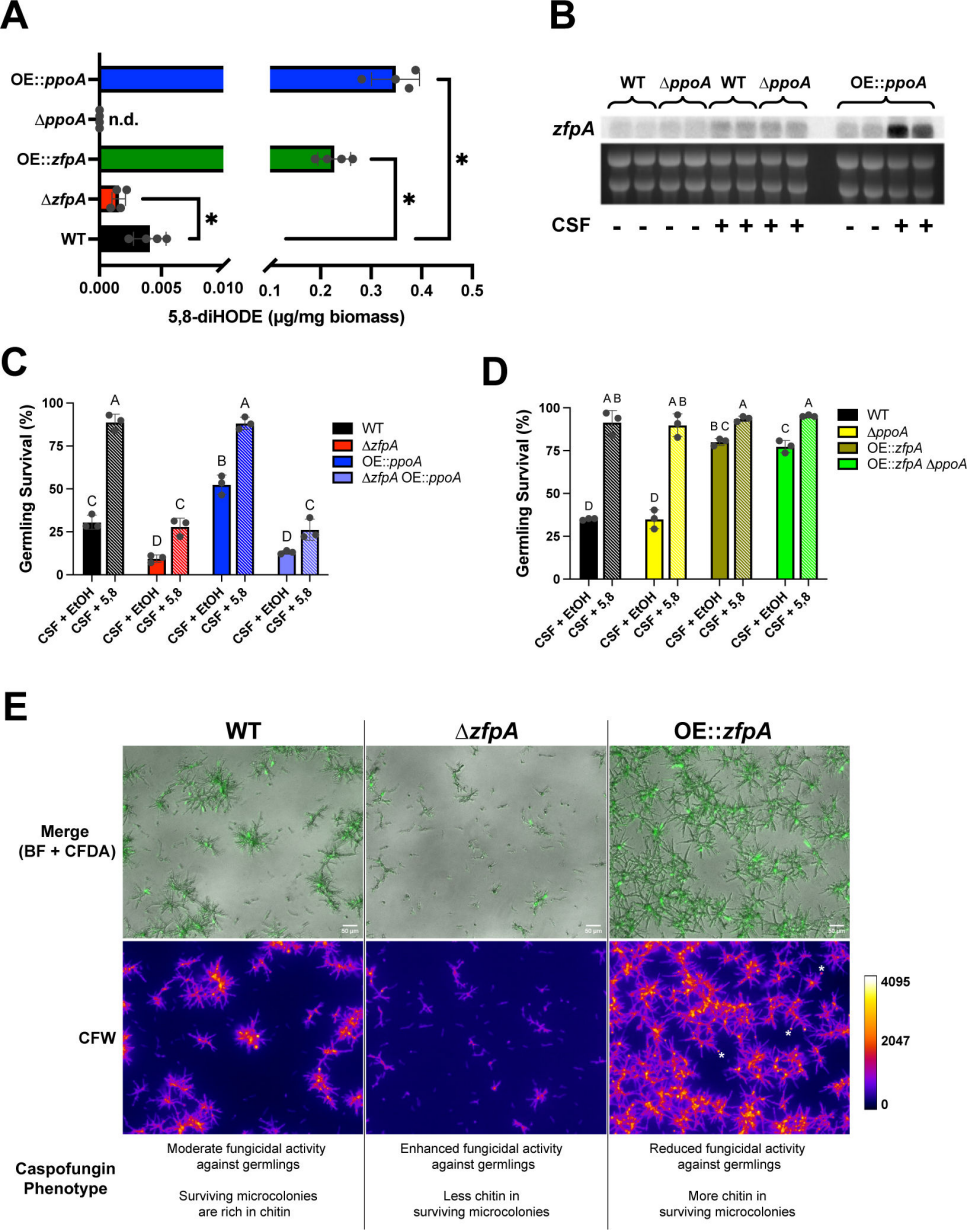
ZfpA and PpoA protect hyphal tips against fungicidal lysis by caspofungin via bidirectional feedback signaling. (**A**) 5,8-diHODE per mg dry biomass extracted from mycelial tissue of *A. fumigatus* Af293. “*” denotes *P* < 0.05 calculated using one-way ANOVA with Tukey’s multiple comparisons after natural log transformation of the data. (**B**) Northern blot analysis of *zfpA* expression in Af293 WT, ∆*ppoA*, and OE::*ppoA* strains under treatment with DMSO vehicle or 2 µg/mL caspofungin (CSF). (**C and D**) Percent survival of *A. fumigatus* Af293 germlings treated with 2 µg/mL caspofungin and 1 µg/mL 5,8-diHODE or EtOH vehicle after 16 h in GMM + 0.01% YE (yeast extract). Conditions with *P* values less than 0.05 calculated by two-way ANOVA with Tukey’s multiple comparisons are indicated by distinct letters. (**E**) Representative micrographs of WT, ∆*zfpA*, and OE::*zfpA* grown for 25 h with 2 µg/mL CSF in the GMM plus 0.01% YE before incubation with 5 (6)-carboxyfluorescein diacetate succinimidyl ester (CFDA-SE) and calcofluor white (CFW) to stain for cellular viability and chitin, respectively, prior to fluorescent and brightfield (BF) imaging.

We have previously shown that exogenous treatment with 5,8-diHODE induces the expression of *zfpA* in *A. fumigatus* (9). In a more recent study, we also showed that ZfpA is required for the induction of *ppoA* expression during treatment with echinocandin drugs (7). Thus, we hypothesized that these two proteins operate in a signaling loop via positive feedback in response to echinocandin stress. To first assess whether ZfpA regulates production of 5,8-diHODE, we quantified 5,8-diHODE in mycelia of *zfpA* deletion and overexpression mutants of *A. fumigatus* Af293. We found that deletion of the *zfpA* significantly reduced the production of 5,8-diHODE compared with the WT fungus, while the overexpression of the gene dramatically increased production, similarly to *ppoA* overexpression (Fig. 1A). We observed similar trends in the production of the 5,8-diHODE precursor (8*R*,9*Z*,12*Z*)-8-hydroxy-9,12-octadecadienoic acid (8-HODE) in the mutants (Fig. S2). Next, we assessed the expression of the *zfpA* transcript in ∆*ppoA* and OE::*ppoA* mutants under treatment with caspofungin or a vehicle control. Northern blot analysis revealed that caspofungin treatment induces *zfpA* expression similarly in the WT and ∆*ppoA* strains but drastically more in the OE::*ppoA* mutant. The OE::*ppoA* strain also expressed *zfpA* more highly than the WT and ∆*ppoA* in the absence of drug treatment (Fig. 1B). Given that ZfpA and 5,8-diHODE both confer protection against echinocandin tip lysis, we generated *zfpA ppoA* double mutants to assess epistasis of these genes in caspofungin tolerance. A ∆*zfpA* OE::*ppoA* double mutant was found to be more sensitive to germling killing by caspofungin tip lysis than the wild type strain, similar to the ∆*zfpA* single mutant, while overexpression of *ppoA* alone conferred only modest protection against tip lysis compared with WT (Fig. 1C). An OE::*zfpA* ∆*ppoA* double mutant was significantly more resistant to fungicidal tip lysis by caspofungin than the WT control but indistinguishable from the OE::*zfpA* single mutant (Fig. 1D). Cotreatment of both double mutants with 5,8-diHODE and caspofungin conferred limited protection against echinocandin tip lysis similar to the respective ∆*zfpA* and OE::*zfpA* single mutants (Fig. 1C and D). These results suggest that ZfpA is required for induction of *ppoA* by caspofungin and the resulting 5,8-diHODE protects against tip lysis via both positive feedback signaling through ZfpA and unknown ZfpA-independent mechanisms. Since *zfpA* was found to be largely epistatic to *ppoA* in tolerance to caspofungin, CFDA-SE staining was used to determine the viability of surviving WT, ∆*zfpA*, and OE::*zfpA* microcolonies following 25-h growth with 2 µg/mL caspofungin treatment in GMM plus 0.01% YE. Subsequently, calcofluor white (CFW) was used to stain the surviving microcolonies to visualize cell wall chitin. Fluorescent microscopy revealed that ∆*zfpA* microcolonies stained less brightly with CFW than WT, whereas the OE::*zfpA* strain produced more chitin-rich microcolonies often exhibiting brightly stained hyphal tips, suggesting higher levels of active chitin synthesis (Fig. 1E). Together, these results suggest that ZfpA is the dominant regulator in a positive feedback loop with PpoA in response to caspofungin treatment, which increases hyphal chitin content.

### Expression of *ppoA* is induced by diverse cell wall stress via ZfpA

**Fig 2:**
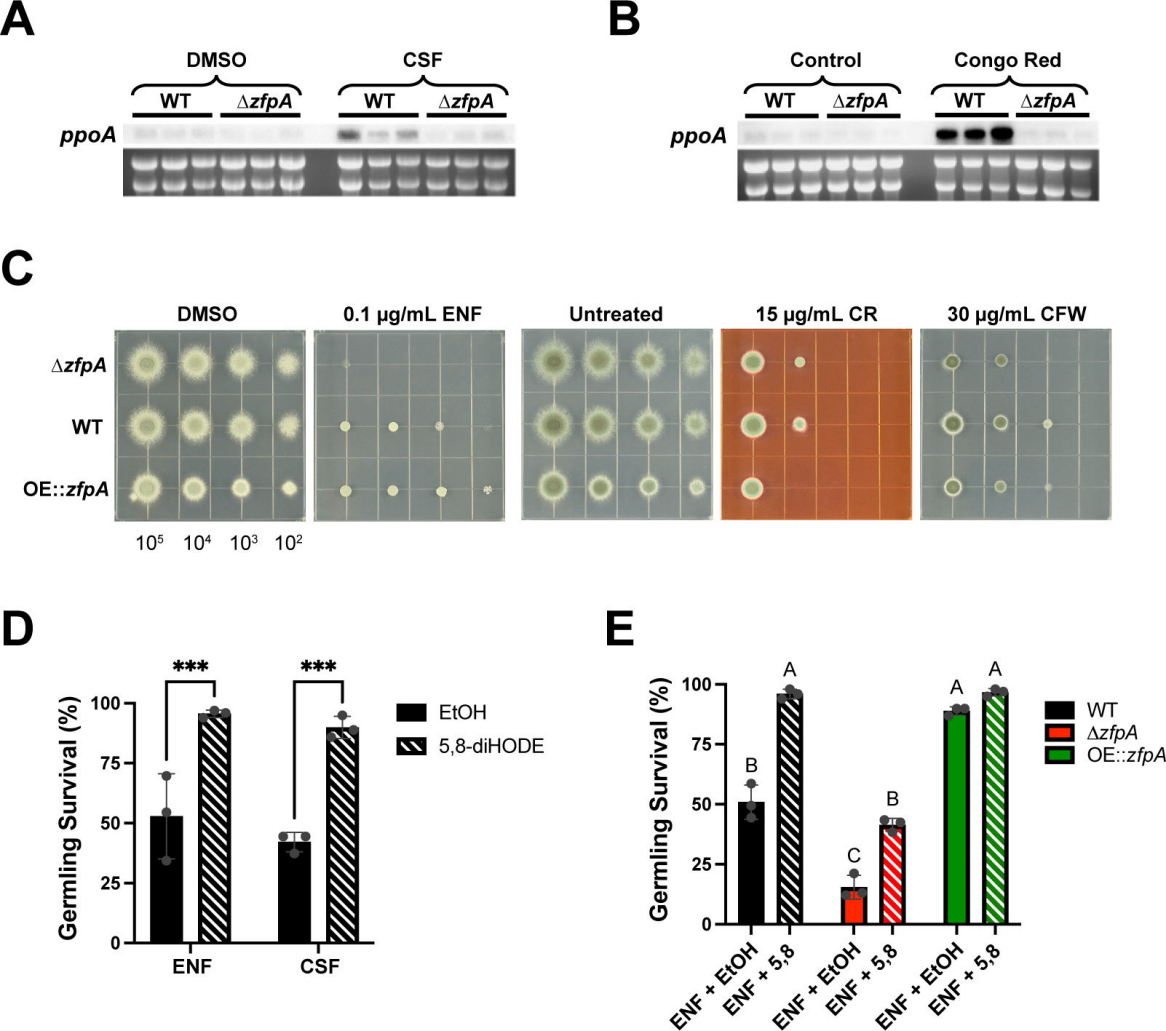
ZfpA/PpoA signaling is induced by diverse cell wall stress agents but specifically protects against β-1,3-glucan synthase-targeting drugs. (**A**) Northern blot analysis of *ppoA* transcript expression in Af293 WT and *∆zfpA* strains under treatment with DMSO vehicle or 2 µg/mL enfumafungin. (**B**) Northern blot analysis of *ppoA* expression in Af293 WT and ∆*zfpA* strains under treatment with vehicle or 50 µg/mL Congo red. (**C**) *A. fumigatus* conidia spotted on GMM after incubation at 37˚C for 48 h. (**D**) Percent survival of *A. fumigatus* Af293 germlings treated with 2 µg/mL caspofungin or enfumafungin (ENF) and 1 µg/mL 5,8-diHODE or EtOH vehicle after 16 h in GMM + 0.01% YE. “***” denotes *P* < 0.001 by Fisher’s LSD test. (**E**) Percent survival of *A. fumigatus* Af293 germlings treated with 2 µg/mL ENF and 1 µg/mL 5,8-diHODE or EtOH vehicle after 16 h in GMM + 0.01% YE. Conditions with *P* values less than 0.05 calculated by two-way ANOVA with Tukey’s multiple comparisons are indicated by distinct letters.

Next, we assessed whether induction of *ppoA* was specific to echinocandin cell wall stress or could be activated more broadly by cell wall perturbation. First, we found that treatment of WT *A. fumigatus* Af293 with enfumafungin, the natural precursor to ibrexafungerp, inhibited hyphal growth in both solid and liquid media more effectively than pneumocandin B_0_, the natural precursor to caspofungin (Fig. S3). Enfumafungin treatment was also found to induce *ppoA* expression in WT *A. fumigatus* strains Af293 and CEA10 (Fig. S4A and B). Furthermore, we found that calcofluor white (CFW) or Congo red (CR) also robustly induced expression of this gene in WT Af293 (Fig. S4C). Northern blotting revealed that *ppoA* expression in response to enfumafungin and Congo red was both ZfpA-dependent, as neither treatment induced the *ppoA* transcript in the ∆*zfpA* mutant (Fig. 2A and B). To evaluate the role of ZfpA in the tolerance of these cell wall-targeting agents, we assayed growth of WT, ∆*zfpA*, and OE::*zfpA* strains on GMM with enfumafungin, CR, or CFW compared with vehicle controls. Dilution plating experiments revealed ZfpA to confer clear protection against enfumafungin, while deletion and overexpression of ZfpA did not appear to significantly impact sensitivity to Congo red or CFW (Fig. 2C). We then assayed the survival of WT Af293 germlings treated with either 2 µg/mL caspofungin or enfumafungin and either 1 µg/mL 5,8-diHODE or ethanol (EtOH) vehicle. The two β-1,3 glucan synthase inhibitors resulted in similar killing of germlings by tip lysis, which was significantly reduced in both cases by 5,8-diHODE cotreatment (Fig. 2D). Protection by 5,8-diHODE against both antifungals was also observed for WT CEA10 (Fig. S5A). Further, we found that ZfpA was required for full protection against tip lysis of germlings under enfumafungin treatment, as the ∆*zfpA* and OE::*zfpA* were poorly protected by 5,8-diHODE cotreatment in both Af293 and CEA10 genetic backgrounds (Fig. 1; Fig. S5B). Together, these data suggest that although diverse cell wall stresses induce a ZfpA/5,8-diHODE signaling response, the activation of this transcription factor only confers specific protection against damage by β-1,3-glucan synthase inhibition.

### Tip lysis due to reduced FksA activity is attenuated by activation of ZfpA

**Fig 3:**
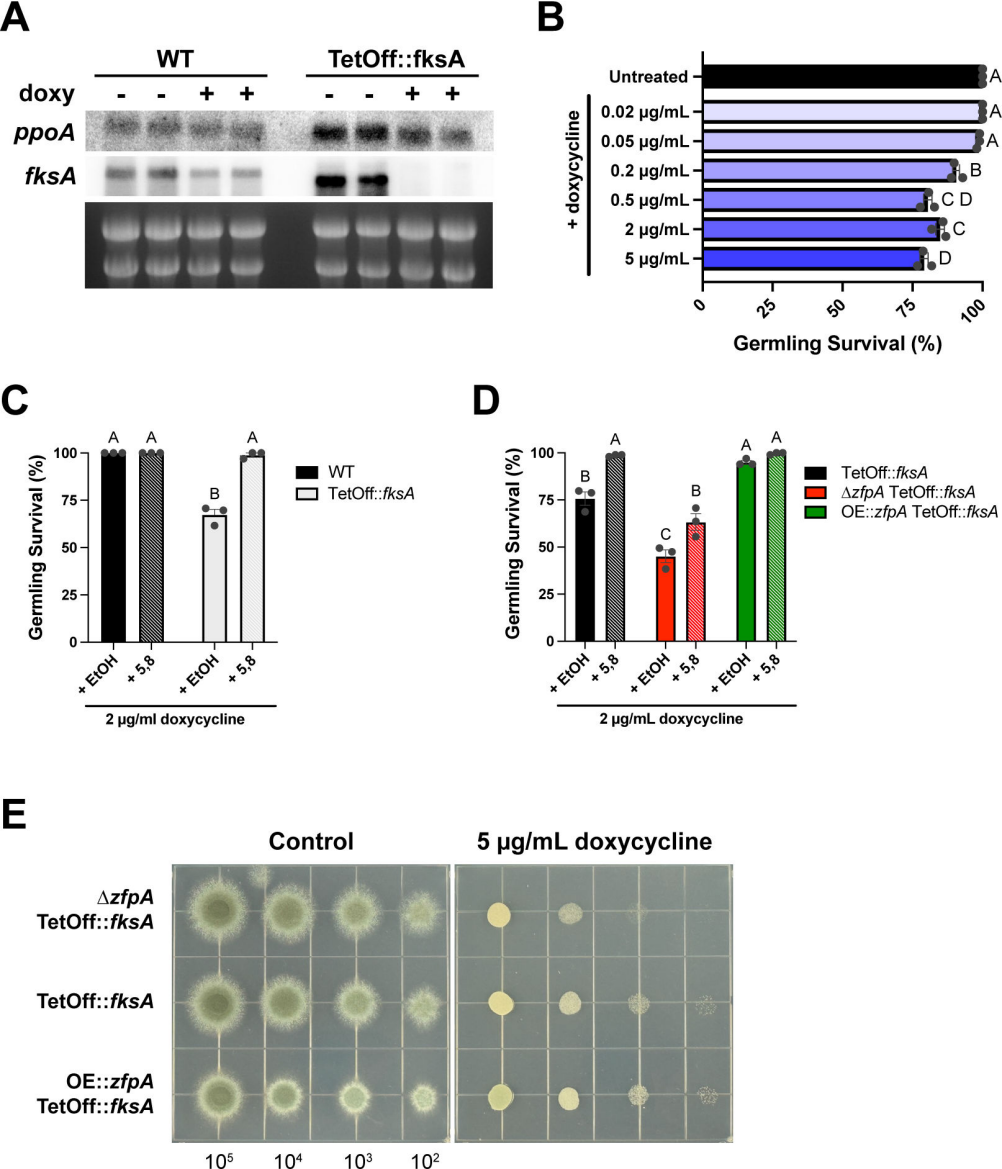
ZfpA and 5,8-diHODE protect against tip lysis due to transcriptional repression of *fksA*. (**A**) Northern blot of *ppoA* and *fksA* genes in *A. fumigatus* Af293 WT and TetOff*::fksA* strains with or without treatment with 5 µg/mL doxycycline (doxy). (**B**) Percent survival of *A. fumigatus* Af293 TetOff::*fksA* germlings treated with increasing concentrations of doxycycline after 16 h in GMM + 0.01% YE. Conditions with *P* values less than 0.05 calculated by one-way ANOVA with Tukey’s multiple comparisons are indicated by distinct letters. (**C**) Percent survival of *A. fumigatus* Af293 WT or TetOff::*fksA* germlings treated with 2 µg/mL doxycycline plus 1 µg/mL 5,8-diHODE or EtOH vehicle after 16 h in GMM + 0.01% YE. Conditions with *P* values less than 0.05 calculated by two-way ANOVA with Tukey’s multiple comparisons are indicated by distinct letters. (**D**) Percent survival of *A. fumigatus* Af293 germlings with 2 µg/mL doxycycline plus 1 µg/mL 5,8-diHODE or EtOH vehicle after 16 h in GMM + 0.01% YE. Conditions with *P* values less than 0.05 calculated by two-way ANOVA with Tukey’s multiple comparisons are indicated by distinct letters. (**E**) *A. fumigatus* Af293 conidia spotted on GMM after incubation at 37˚C for 48 h.

In order to assess whether the activation of 5,8-diHODE/ZfpA by echinocandins and enfumafungin was due to reduced synthesis of β−1,3-glucan by FksA, we generated an *A. fumigatus* Af293 strain expressing the *fksA* gene under a doxycycline repressible promoter using the previously published TetOff system (10) (Fig. S6). While doxycycline treatment did not impact *ppoA* expression in WT *A. fumigatus*, the TetOff::*fksA* strain expressed *ppoA* more highly both under repressive doxycycline levels and in the absence of doxycycline treatment (Fig. 3A). Furthermore, we assessed germling survival of the TetOff::*fksA* mutant in increasing concentrations of doxycycline to inhibit *fksA* expression. We observed that increasing transcriptional repression of *fksA* resulted in increased tip lysis (Fig. 3B), reminiscent of echinocandin/enfumafungin treatment. Further, cotreatment with 5,8-diHODE and doxycycline protected TetOff::*fksA* germlings against killing by tip lysis (Fig. 3C). To determine whether ZfpA was involved, we generated ∆*zfpA* and OE::*zfpA* strains expressing the TetOff::*fksA* construct (Fig. S6). Deletion and overexpression of ZfpA respectively increased and decreased germling killing by tip lysis due to *fksA* transcriptional repression compared with WT (Fig. 3D). Dilution plating of WT, ∆*zfpA*, and OE::*zfpA* strains expressing TetOff::*fksA* on GMM revealed that the deletion of ZfpA also rendered *A. fumigatus* more susceptible to inhibition by *fksA* repression on solid media (Fig. 3E). Together, these results demonstrate that ZfpA is involved in maintaining hyphal tip integrity during reduced β-1,3-glucan synthase activity.

### Four chitin synthases are required for normal chitin synthesis during hyphal growth

**Fig 4:**
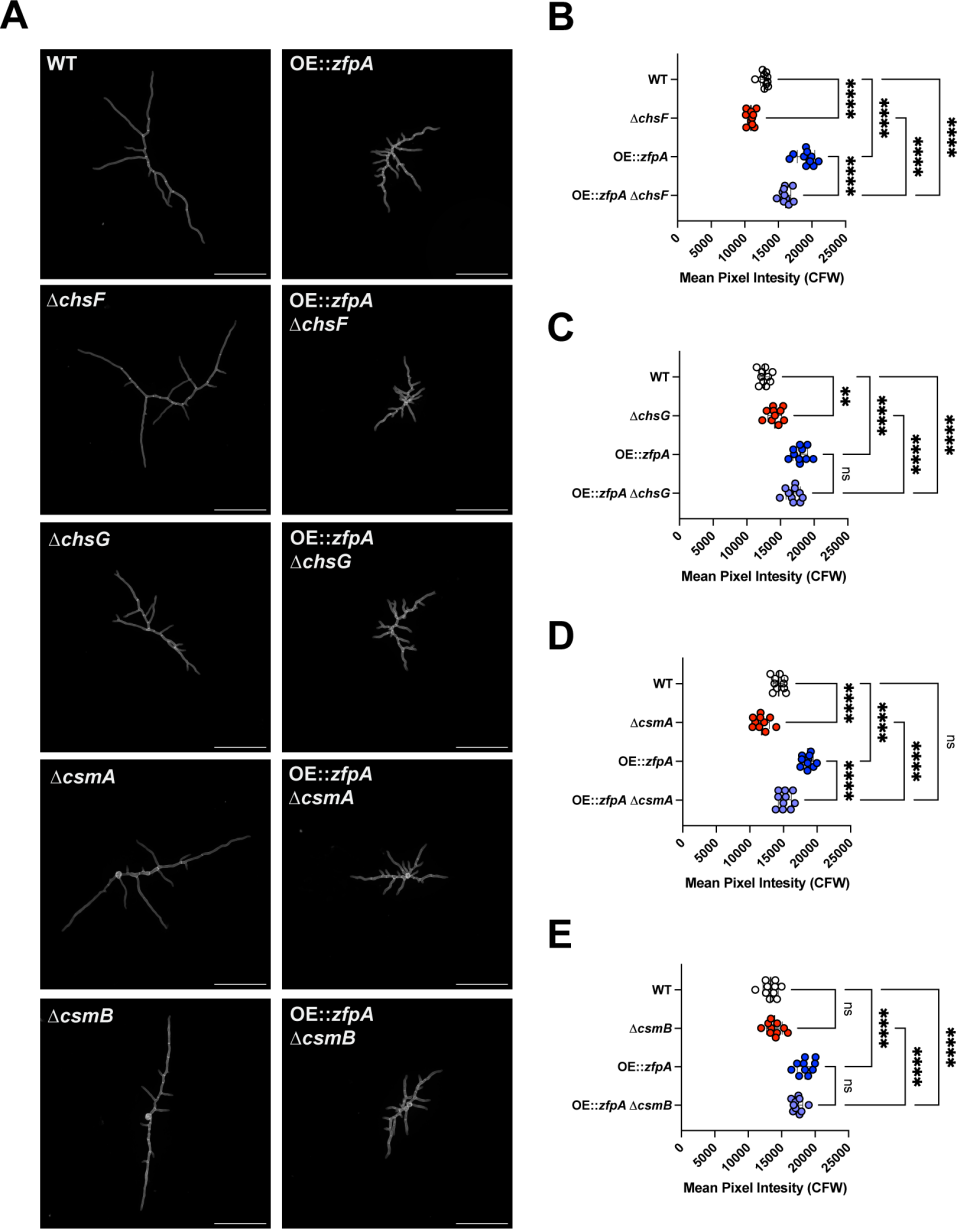
Independent deletions of four chitin synthase genes impact hyphal chitin synthesis in *A. fumigatus*. (**A**) Representative fluorescent micrographs of hyphae grown for 12 h in GMM + 0.01% YE before staining with calcofluor white. Scale bars denote 100 µm. (**B–E**) Mean CFW intensity per pixel of 10 hyphae grown for 12 h in GMM + 0.01% YE before staining and epifluorescent imaging. “**” denotes *P* < 0.01 and “****” denotes *P* < 0.0001 determined by Browne-Forsythe and Welch ANOVA with Dunnett’s T3 multiple comparison tests.

**Fig 5:**
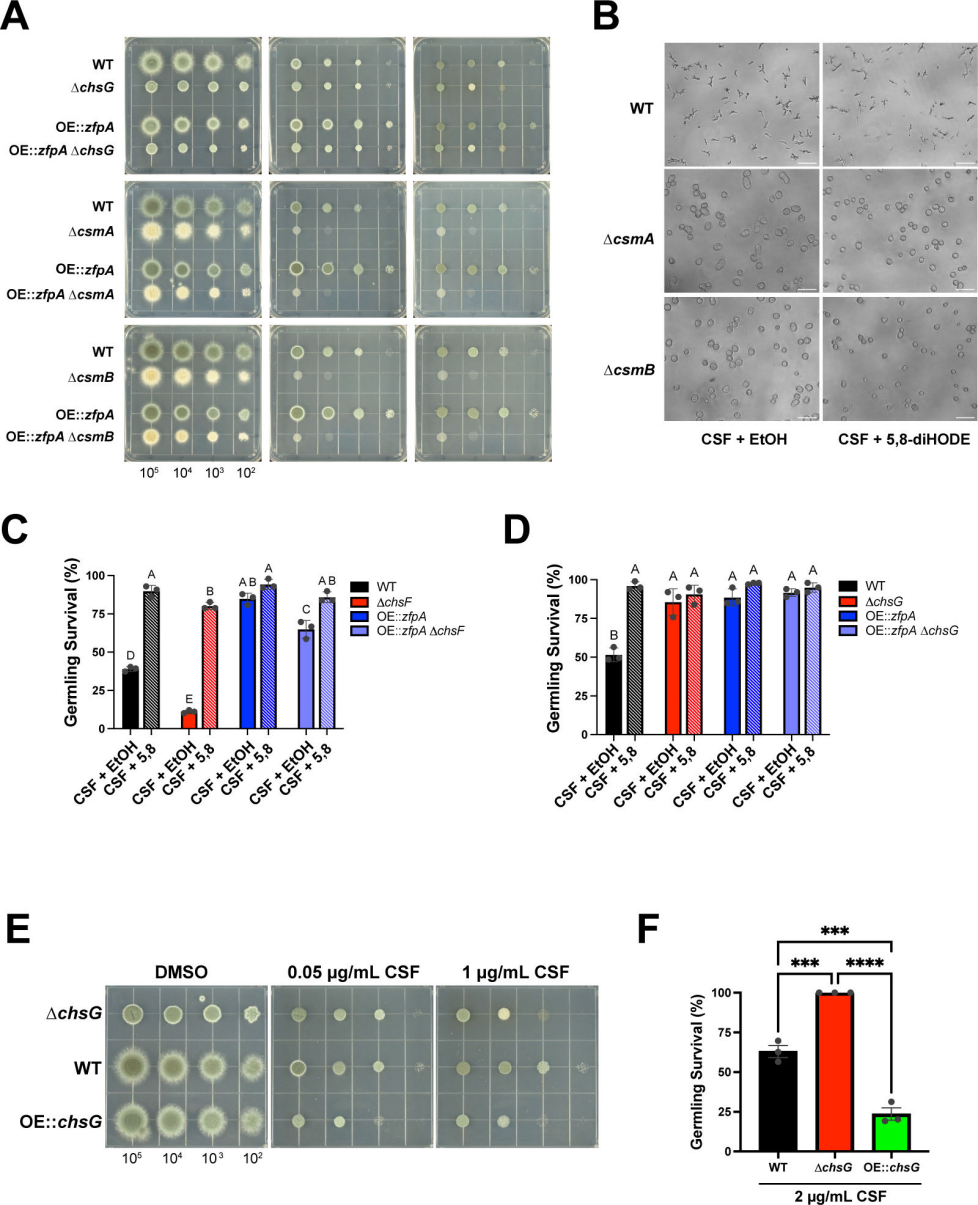
At least four chitin synthases contribute to echinocandin susceptibility in *A. fumigatus*. (**A**) *A. fumigatus* Af293 conidia spotted on GMM after incubation at 37˚C for 48 h. (**B and C**) Percent survival of *A. fumigatus* Af293 germlings treated with 2 µg/mL caspofungin or enfumafungin and 1 µg/mL 5,8-diHODE or EtOH vehicle after 16 h in GMM + 0.01% YE. Conditions with *P* values less than 0.05, calculated by two-way ANOVA with Tukey’s multiple comparisons, are indicated by distinct letters. (**D**) Representative phase micrographs of *A. fumigatus* Af293 conidia incubated for 16 h in GMM + 0.01% YE treated with 2 µg/mL CSF plus 1 µg/mL 5,8-diHODE or EtOH vehicle. Scale bars denote 100 µm. (**E**) *A. fumigatus* conidia spotted on GMM after incubation at 37˚C for 48 h. (**F**) Percent survival of *A. fumigatus* Af293 germlings treated with 2 µg/mL caspofungin after 16 h in GMM + 0.01% YE. “***” denotes *P* < 0.001 and “****” denotes *P* < 0.0001 calculated using one-way ANOVA with Tukey’s multiple comparisons.

It is known that both 5,8-diHODE and echinocandin treatment increase the amount of chitin in the hyphal cell wall in an additive manner, and it has also been demonstrated that ZfpA positively regulates hyphal chitin content (7). Whereas, we previously showed ZfpA to be dispensable in the chitin-synthetic response to caspofungin (11), we found that 5,8-diHODE treatment increased chitin in WT *A. fumigatus* but not the ∆*zfpA* or OE::*zfpA* strains, suggesting that ZfpA mediates the chitin response to this oxylipin (Fig. S7). Thus, we generated single-deletion mutants of each chitin synthase gene in both WT and OE::*zfpA* backgrounds to assess their roles in ZfpA-regulated chitin synthesis (Fig. S8). Hyphal cell wall chitin was assessed in these mutants by CFW staining (Fig. 4). Deletion of *chsA*, *chsB*, *chsC*, or *chsD* alone did not impact chitin levels in either background (Fig. S9). Deletion of *chsF* reduced CFW signal in both the WT and OE::*zfpA* backgrounds (Fig. 4B). Interestingly, deletion of *chsG* in the WT background resulted in a very slight increase in cell wall chitin, which, although statistically significant, may not be biologically conclusive. Loss of ChsG in an OE::*zfpA* background did not impact cell wall chitin (Fig. 4C). Deletion of *csmA* reduced CFW staining in both backgrounds. Total hyphal chitin was not impacted by deletion of *csmB* (Fig. 5E); however, deletion of either *csmA* or *csmB* resulted in an enlarged spore body more brightly stained by CFW (Fig. 5A). Since deletion of *chsF* or *csmA* genes each reduced hyphal chitin in the OE::*zfpA* strain, we assessed expression of their respective transcripts in the ZfpA mutants relative to *gpdA* expression by Northern blot analysis. Neither transcript demonstrated altered expression in the ∆*zfpA* and OE::*zfpA* strains compared with WT, indicating that ZfpA does not directly regulate their expression (Fig. S10). Together, these results suggest that *chsF*, *chsG*, *csmA*, and *csmB* all contribute to cell wall chitin during germination and vegetative growth, but no single chitin synthase is solely responsible for the increased chitin levels seen in the OE::*zfpA* strain.

### Four chitin synthases contribute to tolerance of echinocandins in *A. fumigatus*

Next, we assessed the echinocandin susceptibility of both single and OE::*zfpA* double mutants of all eight chitin synthase genes. Dilution plating revealed that independent deletion of *chsG*, *csmA*, or *csmB* significantly reduced growth of WT and OE::*zfpA* strains on solid GMM with caspofungin treatment (Fig. 5A). However, closer inspection of these mutants under treatment with caspofungin revealed that this was due to impaired filamentation rather than fungicidal activity against these mutants, in agreement with previously published work in the *A. fumigatus* strain CEA10 (12). The deletion of *chsG* drastically reduced colony diameter compared to a WT control on solid GMM with and without caspofungin (Fig. 5A). Deletion of *chsF* did not impact the growth of WT or OE::*zfpA A. fumigatus* on solid media (Fig. S11E). However, the deletion of *chsF* in both backgrounds reduced survival of germlings under caspofungin treatment in liquid media due to increased susceptibility to fungicidal tip lysis (Fig. 5B). Despite apparently reduced growth on solid media, the ∆*chsG* germlings were far more resistant to caspofungin tip lysis than the WT control in liquid media (Fig. 5C). Due to a complete lack of filamentation under caspofungin treatment in liquid media, as was observed on solid media, the ∆*csmA* and ∆*csmB* mutants were not assessed for germling lysis (Fig. 5D). Deletion of *chsA, chsB, chsC*, and *chsD* did not impact growth on solid media or germling survival in liquid media under caspofungin treatment (Fig. S11). Since deletion of *chsG* was found to protect germlings against killing by echinocandin-mediated tip lysis, we were interested to assess whether its overexpression would have an opposite effect. We first generated an OE::*chsG,* which we found to have increased hyphal chitin by CFW staining compared with WT (Fig. S12). Indeed, we found that the OE::*chsG* strain was more sensitive to caspofungin than the WT and ∆*chsG* strains when grown on solid and liquid media, demonstrating increased fungicidal lysis (Fig. 5E and F). Taken together, these data reveal distinct roles for ChsF, ChsG, and CsmA/B in the tolerance of β-1,3-glucan targeting antifungals, with ChsF and ChsG having opposite effects on resistance to fungicidal tip lysis caused by impaired β-1,3-glucan synthesis.

### ChsG impacts sensitivity to fungicidal tip lysis by caspofungin independently of ZfpA and ChsF

**Fig 6:**
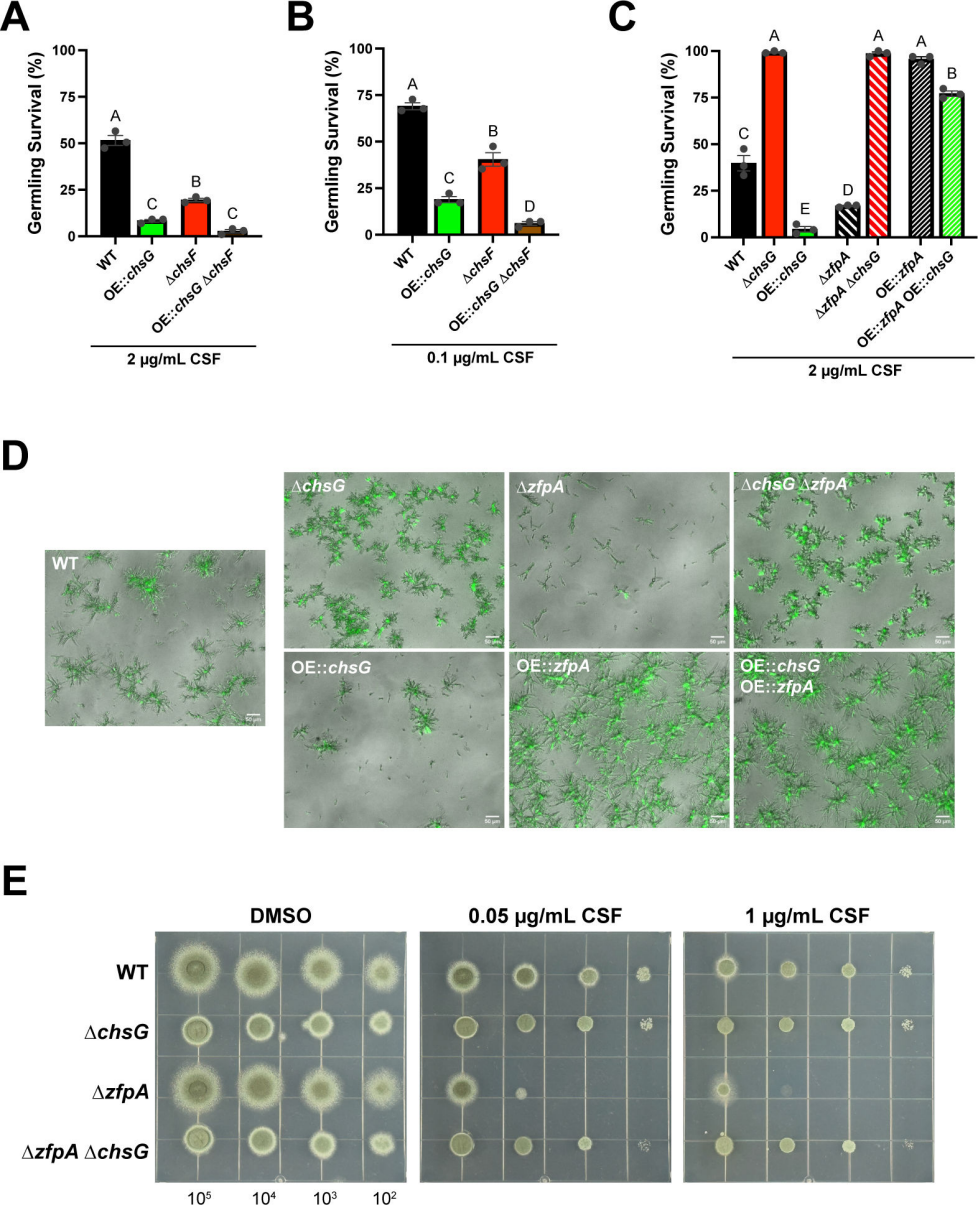
Overexpression of *chsG* increases susceptibility to echinocandins. (**A and B**) Percent survival of *A. fumigatus* Af293 germlings treated with 2 µg/mL (**A**) or 0.1 µg/mL (**B**) caspofungin after 16 h in GMM + 0.01% YE. Conditions with *P* values less than 0.05 calculated by one-way ANOVA with Tukey’s multiple comparisons are indicated by distinct letters. (**C**) Percent survival of *A. fumigatus* Af293 germlings treated with 2 µg/mL caspofungin after 16 h in GMM + 0.01% YE. Conditions with *P* values less than 0.05 calculated by one-way ANOVA with Tukey’s multiple comparisons are indicated by distinct letters. (**D**) Representative micrographs of *A. fumigatus* Af293 WT, ∆*zfpA*, ∆*chsG,* ∆*zfpA* ∆*chsG* double mutant, OE:*chsG,* OE::*zfpA,* and OE:*chsG* OE::*zfpA* double mutant grown for 25 h with 2 µg/mL CSF in GMM plus 0.01% YE before incubation with CFDA-SE for cell viability. Scale bars denote 50 µm. (**E**) *A. fumigatus* Af293 conidia spotted on GMM with corresponding concentrations of caspofungin after incubation at 37˚C for 48 h.

We next generated a double-mutant OE::*chsG* ∆*chsF* strain to assess whether the effects of these chitin synthases were directly related (Fig. S13). At a high concentration, we observed a significant increase in fungicidal lysis of germlings compared to the ∆*chsF* single mutant. The OE::*chsG* ∆*chsF* mutant also showed a trend toward increased lysis compared to the OE::*chsG*, but it did not reach statistical significance (Fig. 6A). However, at a 10-fold lower concentration of caspofungin, we were able to show that loss of ChsF further increased the susceptibility of OE::*chsG* germlings to tip lysis (Fig. 6B). This supports a hypothesis that the two chitin synthases have opposing impacts on maintaining hyphal tip integrity in germlings during β−1,3-glucan inhibition (Fig. 6A and B). To test whether the impact of these two chitin synthases on tolerance to fungicidal tip lysis by caspofungin was dependent on ZfpA, we next generated additional mutants in ∆*zfpA* and OE::*zfpA* backgrounds (Fig. S14). Overexpression of *chsF* did not improve tolerance to fungicidal tip lysis in a WT or ∆*zfpA* background (Fig. S15). However, deletion of *chsG* in the ∆*zfpA* strain phenocopied the ∆*chsG* mutant, exhibiting resistance to caspofungin fungicidal activity but not fungistatic inhibition. Interestingly, the OE::*zfpA* OE::*chsG* double mutant demonstrated an intermediate phenotype between the respective single mutants but was still more tolerant than the WT to fungicidal lysis (Fig. 6C and D). Finally, dilution plating confirmed that *chsG* deletion restored tolerance to the fungicidal activity of caspofungin in the ∆*zfpA* background (Fig. 6E). Taken together, ChsG negatively affects hyphal tip integrity during FksA inhibition, independent of ZfpA-regulated chitin synthesis.

## DISCUSSION

Both ibrexafungerp and the echinocandins are semisynthetic derivatives of secondary metabolites naturally produced by soil-dwelling fungi (13, 14). As has been demonstrated for many microbial secondary metabolites, these compounds may be produced by competing fungi in shared environmental niches as a means of antimicrobial warfare. As a ubiquitous saprophyte, *A. fumigatus* also inhabits the soil niche. Thus, it is possible that sustained competition with fungal species producing β-1,3-glucan synthase-inhibiting compounds, such as enfumafungin and echinocandins, resulted in evolved mechanisms of tolerance against these antifungal metabolites. Here, we describe one such response mediated by the transcription factor ZfpA and the associated 5,8-diHODE producing enzyme PpoA, which confers protection against fungicidal tip lysis caused by β-1,3-glucan synthase inhibition, in part by changes in hyphal chitin synthesis.

It has been previously shown that the oxylipin 5,8-diHODE protects against fungicidal tip lysis by the echinocandin antifungals. Additionally, it was demonstrated that the C2H2 transcription factor ZfpA is required for the induction of this oxylipin signal and is the central mediator of the protective response to it (7). Our findings that overexpression and deletion of *zfpA,* respectively, increase and decrease 5,8-diHODE production, strengthen prior results showing that ZfpA regulates transcriptional expression of *ppoA*. Furthermore, we confirmed that a positive feedback loop exists between these two proteins, in that the overexpression of *ppoA* also increases *zfpA* expression, especially during caspofungin treatment. However, assessment of caspofungin tip lysis in *zfpA ppoA* germlings revealed that the former is largely epistatic to the latter with regard to echinocandin susceptibility. This is likely due to the dual function of ZfpA in both regulating the production of 5,8-diHODE and directing the transcriptional response to this signal. However, some ZfpA-independent protection against echinocandin tip lysis is still supported by modest protection observed in the ∆*zfpA* strains in the presence of exogenous 5,8-diHODE cotreatment. This may be mediated by other transcription factors previously identified to be responsive to this oxylipin (9).

While the cell wall-targeting echinocandins caspofungin and micafungin were reported to induce 5,8-diHODE synthesis in *A. fumigatus*, the membrane-targeting antifungal voriconazole did not (7). Therefore, we sought to determine whether this ZfpA/oxylipin signaling response was specific to echinocandins or if it could be induced by other types of cell wall stress. Both enfumafungin, the natural precursor to ibrexafungerp, and ibrexafungerp itself, are triterpenoids that exhibit antifungal activity similar to the echinocandins (15). However, the binding site of these compounds and the echinocandins only partially overlap (16). We found that *ppoA* expression was induced similarly by enfumafungin, the natural echinocandin pneumocandin B_0_, and its semisynthetic derivative caspofungin in both *A. fumigatus* Af293 and CEA10 isolates. Published RNA sequencing data suggest that treatment with the cell wall-perturbing agent Congo red also induces *zfpA* expression (17). Therefore, we assessed the downstream expression of *ppoA* under treatment with this agent as well as under treatment with growth-inhibitory levels of calcofluor white. Indeed, we found that both cell wall-perturbing agents also induced *ppoA* expression. As previously described for echinocandins, the induction of *ppoA* in response to enfumafungin and Congo red required functional ZfpA, as the *ppoA* transcript was not upregulated in the ∆*zfpA* mutant. Together, these data suggest that cell wall perturbation broadly activates 5,8-diHODE signaling via ZfpA, possibly through the known cell wall integrity stress sensors Wsc1, Wsc2, and MidA (18). However, further investigation is necessary to confirm the upstream signaling responsible for ZfpA activation during cell wall stress. Importantly, dilution plating of *zfpA* deletion and overexpression mutants revealed that ZfpA mediates tolerance of enfumafungin but not Congo red or calcofluor white. This contrasts prior work showing deletion of *zfpA* to increase sensitivity of *A. fumigatus* CEA10 to CR and CFW (9, 11). We suspect this is a result of differences in cell wall architecture between the two clinical isolates CEA10 and Af293. Further experimentation showed that 5,8-diHODE protects against fungicidal tip lysis by enfumafungin in a ZfpA-dependent manner, similar to prior findings with echinocandin drugs (7). These data suggest that ZfpA regulates cell wall synthetic processes that stabilize the growing tip to prevent lysis during β-1,3-glucan synthase inhibition.

To demonstrate that the protective ZfpA/5,8-diHODE signaling response to these antifungals is a direct result of reduced FksA activity, we used a genetic approach to decrease β-1,3-glucan synthesis by replacing the native *fksA* promoter with a tetracycline/doxycycline repressible promoter. Treatment with 2 µg/mL doxycycline completely abolished detectable transcript in the TetOff::*fksA* strain. Interestingly, expression of *fksA* in this mutant in the absence of doxycycline treatment was higher than expression under the native promoter. This may explain why *ppoA* expression was found to be increased in the TetOff::*fksA* strain compared with WT both in the presence and absence of doxycycline treatment. This result reinforces the conclusion that any significant perturbation to normal cell wall architecture can induce ZfpA/5,8-diHODE signaling. Reduced FksA activity due to transcriptional repression also resulted in increased tip lysis as measured by germling survival, as previously observed under pharmacologic inhibition of FksA activity. Germling survival during *fksA* repression was improved by 5,8-diHODE treatment, reducing the lysis of growing tips, and this protection was found to be largely ZfpA-dependent. These findings agree with published work by Loiko and Wagener, who found that the susceptibility of *A. fumigatus* strain AfS35 to fungicidal tip lysis by caspofungin was directly correlated with *fksA* expression (19).

Prior studies suggest that an upregulation of chitin can confer protection against echinocandin drugs, especially during early exposure (20–22). This is hypothesized to be the result of structural stabilization by compensatory chitin in the absence of sufficient β-1,3-glucan. It was also shown that both 5,8-diHODE and caspofungin increase cell wall chitin in *A. fumigatus* hyphae (7). However, the increased chitin in response to caspofungin did not require ZfpA (11). In contrast, we show that increased hyphal chitin in response to 5,8-diHODE is entirely ZfpA-dependent, as treatment with this oxylipin failed to increase hyphal chitin in either the ∆*zfpA* or OE::*zfpA* mutant. This suggests that the chitin synthetic response to 5,8-diHODE is likely distinct from that of the echinocandins. Caspofungin treatment was reported to induce the expression of several chitin synthases—*chsA*, *chsG*, and *csmB*—most highly (23). Conversely, RNA-sequencing of *A. fumigatus* in response to 5,8-diHODE was not reported to significantly alter the expression of any chitin synthase genes (9). However, it is possible that ZfpA alters hyphal chitin levels indirectly by regulating the expression of yet unidentified genes encoding proteins that modulate the localization, activity, or function of certain chitin synthases.

Although the distinct enzymatic functions of the eight chitin synthases in *A. fumigatus* are not clearly defined, it is known that these enzymes can be activated by interactions with associated proteins (8, 24). Therefore, to further assess the regulatory role of ZfpA in chitin synthesis, we independently deleted each chitin synthase gene in WT and OE::*zfpA* backgrounds. Deletion of *chsA*, *chsB*, *chsC*, and *chsD* did not significantly impact cell wall chitin detectable by CFW staining of *A. fumigatus* hyphae in either background, but deletion of *chsF*, *chsG*, *csmA,* or *csmB* did. First, deletion of *chsF* reduced total chitin in a WT background, suggesting that it is involved in chitin synthesis during vegetative growth. Deletion in an OE::*zfpA* background also reduced total chitin, but the OE::*zfpA* ∆*chsF* double mutant still showed higher levels of chitin than the ∆*chsF* mutant, suggesting that ChsF activity alone does not account for the ZfpA-dependent increase in chitin. Interestingly, deletion of *chsG* in a WT background slightly increased hyphal chitin, suggesting a possible compensatory response by other chitin synthases. However, this small increase may not be biologically significant, considering that CFW staining is an imperfect method for the quantification of chitin due to its binding to chitosan as well as incomplete chitin fragments. Deletion of *chsG* in an OE::*zfpA* background had no impact on CFW staining, suggesting that the overexpression of ZfpA and deletion of *chsG* may both increase the activity of other chitin synthases. CsmA and CsmB are unique from the other chitin synthases in that they each contain an N-terminal myosin motor domain (12). This myosin motor domain in a homologous enzyme of *U. maydis* has been shown to be important for proper fusion of chitin synthase-containing vesicles at hyphal tips (25, 26). This function is likely important for proper polarization during filamentous growth, as deletion of either *csm* gene resulted in abnormally enlarged spore bodies that stained more brightly with CFW. Uniquely, the deletion of *csmA* in an OE::*zfpA* background returned hyphal chitin to wild-type levels. This may suggest that CsmA activity is increased under ZfpA overexpression, although we did not find the expression of the *csmA* to be significantly altered by Northern blot analysis under the conditions tested. Taken together, this suggests that ChsF, ChsG, CsmA, and CsmB are all required for normal chitin synthesis during germination and hyphal growth.

Next, we assessed caspofungin growth inhibition and tip lysis in the deletion mutants of the eight chitin synthases. In agreement with published work with an ∆*chsA* ∆*chsC* double mutant in an Af293 background (27), we found no impact of *chsA*, *chsB*, *chsC*, or *chsD* deletion on caspofungin susceptibility. Prior work with *A. fumigatus* A1160 did not reveal a role for ChsA, ChsB, ChsC, ChsD, or ChsF in caspofungin tolerance on solid minimal media (28). While we also saw no difference in radial growth compared with WT of the ∆*chsF* strain on solid media with caspofungin, microscopic assessment of germling survival in liquid media revealed increased echinocandin tip lysis in this mutant. Increased tip lysis was also observed in the OE::*zfpA* ∆*chsF* double mutant compared with an OE::*zfpA* control. This suggests that ChsF is important for resistance to fungicidal tip lysis by β-1,3-glucan targeting antifungals but not their fungistatic effects. Deletion of *chsG* in a WT strain resulted in reduced radial growth on solid media, in agreement with prior findings in an A1160 background (28). Conversely, we found the deletion of this gene in a WT Af293 background to reduce fungicidal tip lysis by caspofungin, as was observed for the OE::*zfpA* strain. This supports a potential hypothesis that similar chitin synthetic pathways are activated in these two mutants, which stabilize the growing tip in the absence of β-1,3-glucan. However, it is also possible that other cell wall components contribute to hyphal tip integrity, and it remains to be determined which specific enzymes are involved. It has been previously reported that deletion of *csmA* and *csmB* dramatically impairs hyphal growth and increases caspofungin susceptibility (12). Indeed, we found that deletion of either gene resulted in abnormal hyphal growth, poor conidiation, and hypersensitivity to caspofungin in both WT and OE::*zfpA* backgrounds. However, microscopic imaging of these strains revealed that the inhibition by caspofungin is the result of an inability for swelling conidia to switch to polar growth. Germinating conidia of ∆*csmA* and ∆*csmB* mutants treated with caspofungin failed to properly polarize to form germ tubes as shown previously in the strain A1160 (28). Due to the absence of normal germ tube, these mutants were not assessed for germling lysis, but phase microscopy revealed that they continued to swell isotropically out to 24 h. This indicates that both functional CsmA and CsmB, as well as β-1,3-glucan synthase activity, are required for filamentous growth in *A. fumigatus*. Together, these results suggest that ChsF, CsmA, and CsmB are involved in echinocandin tolerance. While ChsF confers tolerance to fungicidal tip lysis by echinocandins, CsmA, and CsmB are necessary for resistance to the fungistatic inhibition by these antifungals. Uniquely, deletion of ChsG simultaneously impairs hyphal growth on solid media and confers resistance to tip lysis by echinocandins. Given the opposite phenotypes of the ∆*chsF* and ∆*chsG* in caspofungin tip lysis, it is possible that loss of ChsG results in increased ChsF activity, which stabilizes the growing tip. However, it cannot be ruled out that altered activity of the other six chitin synthases may also reinforce the growing tip during FksA inhibition.

Since deletion of *chsG* uniquely impaired filamentous growth on solid GMM but improved germling survival under treatment with caspofungin in liquid media, we were interested to assess whether overexpression of this gene would have the opposite effect. Indeed, the finding that overexpression of *chsG* increased susceptibility of *A. fumigatus* to caspofungin treatment, both in liquid and solid GMM, supports a negative role for this chitin synthase in stabilization of the growing tip during β-1,3-glucan depletion. Since deletion of *chsF* also rendered germlings more susceptible to growing tip lysis by caspofungin, we sought to test whether an OE::*chsG* ∆*chsF* double mutant would be even more susceptible to tip lysis than either alone. Indeed, we found that the OE::*chsG* ∆*chsF* germlings were extremely susceptible to killing by caspofungin tip lysis, with significantly reduced survival compared with both single mutant strains. These findings support independent yet opposing functions of these chitin synthases in stabilizing the growing tip during reduced β1,3-glucan synthesis.

Finally, we sought to assess whether the impacts of ChsF and ChsG on the fungicidal effect of caspofungin in germlings were dependent on ZfpA by generating additional double mutants in the ∆*zfpA* and OE::*zfpA* backgrounds. Overexpression of *chsF* did not improve survival of ∆*zfpA* germlings, suggesting it is not directly regulated by ZfpA, but this does not rule out indirect regulation of its expression or activity at the protein level by ZfpA. In contrast, deletion of *chsG* completely protected ∆*zfpA* germlings against fungicidal tip lysis. Simultaneous overexpression of both *chsG* and *zfpA* had a mixed phenotype, most closely resembling the OE::*zfpA* strain. Based on these results, ZfpA-regulated chitin synthesis is important for maintaining the integrity of growing tips during β-1,3-glucan synthase inhibition, but identification of the contribution of specific chitin synthase enzymes will likely require more advanced chemical analyses.

This study reveals that the previously described ZfpA/5,8-diHODE signaling response is not specific to echinocandins but rather induced broadly by disruption of the normal cell wall architecture. However, the protection conferred by this oxylipin-associated transcription factor is specific to only β-1,3-glucan depletion, protecting growing tips against fungicidal lysis. Interestingly, both enfumafungin and natural echinocandins are produced by other soil-dwelling ascomycetes. As has been described for other secondary metabolites, it is likely that these antifungal compounds evolved as a mechanism of antimicrobial warfare against competing fungi in a shared environmental niche. As *A. fumigatus* is also found abundantly in soil, repeated encounters with these antifungal-producing fungi may have led to the development of this ZfpA-mediated protective response. Here, we found that at least four chitin synthases impact echinocandin susceptibility, with ChsF and ChsG having opposing impacts on the structural integrity of β-1,3-glucan-depleted hyphal tips. While ZfpA-dependent increases in cell wall chitin cannot fully explain the protection by 5,8-diHODE against FksA-targeting antifungals, chitin and cell wall reorganization are one part of what is likely a multifactorial protective response coordinated by the transcription factor ZfpA.

## MATERIALS AND METHODS

### Fungal strains, media, and culture conditions

Wild-type *A. fumigatus* Af293 and CEA10 are common clinical laboratory strains available through ATCC. All fungal strains used in this study are described in Table S1. Strains were maintained in 25%–50% glycerol stock suspensions at −80°C and grown on glucose minimal media (GMM) plates at 37°C for 3 days. Conidia were collected into sterile water with 0.01% Tween 80. GMM plates and broth were prepared, as previously described (29).

### Strain construction and southern blotting

To complement *A. fumigatus argB* and *pyrG* in *A. fumigatus*, the *argB* or *pyrG* locus was amplified from WT Af293 with 1 kb 5′ and 3′ flanks. The parental auxotroph was transformed following the previously described approach (30). To generate gene deletion strains of *A. fumigatus*, two 1-kb DNA fragments immediately upstream and downstream of each open reading frame (ORF) were amplified by PCR from WT Af293 genomic DNA and fused to a 2-kb *A. parasiticus pyrG* or *A. fumigatus argB* fragment from pJW24 or WT Af293 gDNA using double joint PCR (31, 32). Transformants were confirmed by Southern blotting using restriction enzyme digest with both dCTP-αP^32^ labeled 5′ and 3′ flanks of each knockout construct. To generate overexpression constructs, two 1-kb fragments immediately upstream and downstream of the translational start site were amplified by PCR from *WT A. fumigatus* Af293 genomic DNA and *A. parasiticus pyrG::A. nidulans gpdA(P*) or *A.* fumigatus *argB:: A. nidulans gpdA(P)* as the selectable marker and overexpression promoter were amplified from the plasmids pJMP9 or pJMP10 (33). These three fragments were fused by double joint PCR. To generate doxycycline-inducible promoter constructs, two 1-kb fragments immediately upstream and downstream of the translational start site were amplified by PCR from *WT A. fumigatus* Af293 genomic DNA, and PtrA::TetOff(*P*) as the selectable marker and inducible promoter was amplified from the plasmids pCH008 (33). The three fragments were fused by double joint PCR. Constructs were transformed into the specified parental strains as previously described. Single integration of the transformation construct was confirmed by Southern blotting using restriction enzyme digest with both dCTP-αP^32^ labeled 5′ and 3′ flanks of each overexpression construct.

### Fungal oxylipin extraction

*A. fumigatus* WT Af293 conidia were inoculated at 1 × 10^6^ spores/mL into 50 mL GMM and incubated at 37°C and 250 RPM for 48 h. Supernatants were filtered into 250-mL glass bottles by collecting mycelial tissue into sterile miracloth. The collected tissue was washed with milli-Q water, press dried, frozen, and lyophilized. Supernatants were extracted by combining with 50 mL ethyl acetate and shaking in a separatory funnel. The organic phase was collected and evaporated to dryness. Lyophilized tissue was weighed to determine dry biomass before being homogenized in 5 mL sterile milli-Q water. Homogenized tissue was extracted overnight in 100 mL ethyl acetate. Tissue and debris were filtered out using Whatman paper filters, and equal volume milli-Q water was added to the organic solvent in a separatory funnel. The organic layer was collected after shaking and venting twice, and evaporated to dryness. Total extracts were dissolved in 4 mL of methanol (MeOH) and transferred to pre-weighed 20-mL glass scintillation vials and evaporated to dryness again. Dried extracts were weighed and stored at −80°C. Extracts were dissolved at 1 mg/mL in MeOH for analysis by UHPLC–HRMS/MS.

### UHPLC–HRMS/MS analysis

Ultra high pressure liquid chromatography–high resolution tandem mass spectrometry (UHPLC–MS/MS) data were acquired using a Thermo Scientific Q Exactive Orbitrap mass spectrometer coupled to a Vanquish UHPLC operated in both positive and negative ionization modes. All solvents used were of spectroscopic grade. Each sample was filtered with 0.2-µm syringe filter. A Waters XBridge BEH-C18 column (2.1 × 100 mm, 1.7 μm) was used with acetonitrile (0.1% formic acid) and water (0.1% formic acid) as solvents at a flow rate of 0.2 mL/min. The screening gradient method for the samples is as follows: starting at 55% organic, hold for 1 min, followed by a linear increase to 98% organic over 18 min, holding at 98% organic for 2 min, for a total of 21 min. A quantity of 10 μL of each sample was injected into the system for analysis. Purified 5,8-diHODE and 8-HODE were used as standards. For quantification, standard curves for 5,8-diHODE and 8-HODE were calculated based on intensities from six different concentrations of each purified oxylipin (5, 2.5, 1.25, 0.625, 0.3125, and 0.15625 ppm).

### Antifungal drugs and oxylipins

All antifungals, including caspofungin, micafungin, and enfumafungin, were purchased from ApexBio and stored as stock concentrations in DMSO at −20°C. Purified fungal oxylipins 5,8-diHODE and 8-HODE were prepared as previously described (34). Oxylipin stocks were stored at −20°C in EtOH.

### RNA extraction and northern blotting

Conidia were inoculated at 10^6^ spores/mL into 50 mL GMM and incubated at 37°C and 250 RPM. Fungal cultures for *ppoA* expression were incubated for 18.5 h before addition of drug or vehicle control for an additional 90 min. Cultures for chitinsynthase gene expression were incubated for 24 h. Tissue was collected into sterile miracloth, flash frozen, and lyophilized. Total RNA was extracted using QIAzol lysis reagent (Qiagen) per manufacturer’s protocol with the addition of a phenol:chloroform:isoamyl alcohol (25:24:1) extraction step before RNA precipitation. RNA purity and concentration were assessed by nanodrop. Approximately 12 µg of each sample was run in a 1.1% agarose 1.5% formaldehyde gel and transferred to an Amersham Hybond *N*^+^ Membrane. Membranes were hybridized with a doubly labeled dCTP-αP^32^ dATP-αP^32^ probe complementary to a 500-bp to 1-kb region of the gene of interest lacking any predicted introns.

### Germling lysis experiments

For each experimental condition, approximately 4,000 conidia were inoculated into three independent wells of a 48-well plate in 0.4 mL GMM broth with 0.01% w/v yeast extract. The plate was incubated on a Nikon Eclipse Ti Inverted Microscope in a heated microscope enclosure (OKO Labs, Burlingame, CA) at 37°C for 4 h before imaging. During this pre-incubation, three XY imaging positions were set in each well with at least 33 visible spores in each frame. Images were acquired every fifteen minutes at each XY position for 20 h using a Nikon Plan Fluor 10X Ph1 DLL objective. Ninety-nine spores from the three frames in each well were selected blindly at time zero (4 h post inoculation) and annotated in NIS-Elements AR software package (Version 5.30) to be assessed for lysis at 16 h post inoculation. The number of annotated germlings lysed by 16 h post inoculation was manually counted for each of the three wells per condition. Complete lysis of germlings was determined by the loss of normal diffraction of light compared to intact hyphae and confirmed by complete cessation of growth out to 24 h, as previously described (7). Endpoint viability and chitin of microcolonies were determined by staining with 40 µg/mL CFDA-SE and 0.1 mg/mL CFW respectively.

### Stress test dilution plating

To assess cell wall stress tolerance, 30-mL GMM square plates with the specified drug treatment or vehicle controls were inoculated with 10^5^, 10^4^, 10^3^, and 10^2^ spores of each strain and grown at 37°C for 48 h. All experiments were completed in triplicate with representative pictures presented in figures.

### CFW cell wall chitin quantification

In total, 1,000 conidia of WT *A. fumigatus* Af293 in 1 mL GMM + 0.01% YE were inoculated into wells of a coverslip glass bottom 24-well plate. After incubation at 37°C for 12 h, the hyphae were rinsed once with PBS before staining for 5 min with calcofluor white solution (Sigma-Aldrich). Stained hyphae were washed twice for 5 min with fresh PBS before DAPI channel fluorescent imaging on a Nikon Ti2E inverted microscope with a Nikon CFI Plan Apochromat Lambda D 20× objective. Mean CFW signal intensity per pixel of 10 hyphae per condition was measured using FIJI (v2.14.0/1.54f) by using the auto-thresholding and wand tracing tool functions to select the hyphal area before quantifying mean pixel intensity, as previously described (7).

## SUPPLEMENTAL MATERIAL

Supplemental material aac.01769-25-s0001.pdf Fig. S1 to S15; Tables S1 and S2.

ASM does not own the copyrights to Supplemental Material that may be linked to, or accessed through, an article. The authors have granted ASM a non-exclusive, world-wide license to publish the Supplemental Material files. Please contact the corresponding author directly for reuse.
