# Supplementary material for: ZfpA-regulated chitin synthesis in Aspergillus fumigatus hyphae determines fungicidal tip lysis by FksA-targeting antifungals
Source: Antimicrob Agents Chemother. 2026 Jun 4;70(7):e01769-25. doi: 10.1128/aac.01769-25 (PMC13321839; doi:10.1128/aac.01769-25)
Supplement: Supplemental material — Fig. S1 to S15; Tables S1 and S2. [file aac.01769-25-s0001.pdf]

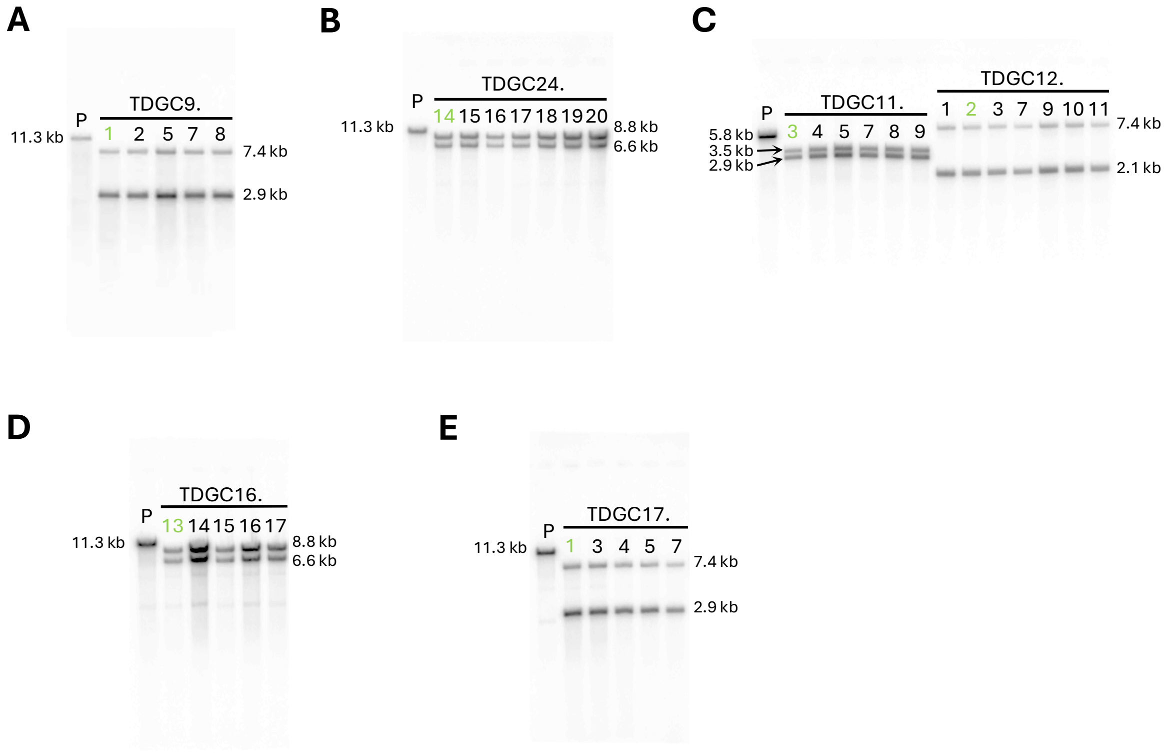

Figure S1. Southern blot confirmation of PpoA and ZfpA mutant strains. (A) Southern blot of FspI digested genomic DNA of parental strain TFYL45.1 and Δ*ppoA* mutants. **(B)** Southern blot of FspI digested genomic DNA of parental strain TDGC19.1 and OE::*ppoA* mutants. **(C)** Southern blot of Nsi-HF digested genomic DNA of parental strain TFYL45.1 and Δ*zfpA* and OE::*zfpA* mutants. **(D)** Southern blot of FspI digested genomic DNA of parental strain TDGC11.3 and OE::*ppoA* mutants. **(E)** Southern blot of FspI digested genomic DNA of parental strain TDGC12.2 and Δ*ppoA* mutants.

A

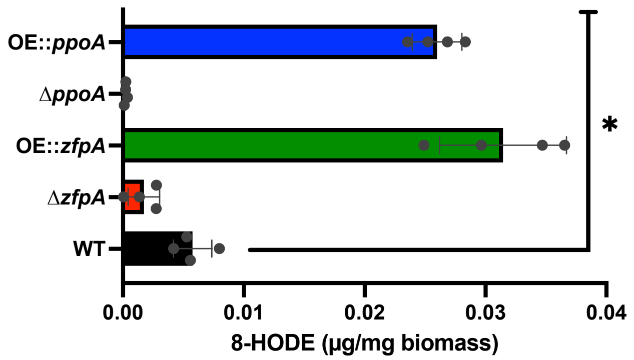

Figure S2. ZfpA regulates production of 8-HODE in A. fumigatus Af293. (A) 8-HODE per mg dry biomass extracted from mycelial tissue of *A. fumigatus* Af293. * Denotes $p < 0.0125$ when compared to wild type calculated using two-sided Welch's t-test with Bonferroni correction.

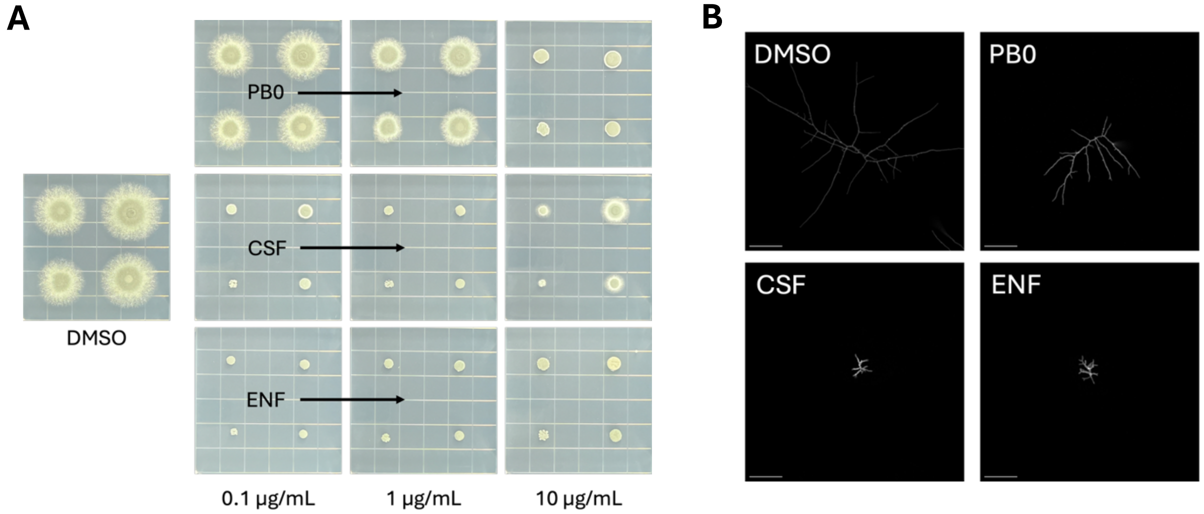

Figure S3. FksA targeting antifungal compounds inhibit growth of *A. fumigatus*. (A) WT *A. fumigatus* Af293 grown on solid GMM with pneumocandin B0, caspofungin, or enfumafungin for 48 hours at 37˚C. (B) Example micrographs of WT *A. fumigatus* Af293 hyphae stained with CFW after 24 hours growth at 37˚C in liquid GMM with 1 µg/mL pneumocandin B0, caspofungin, or enfumafungin.

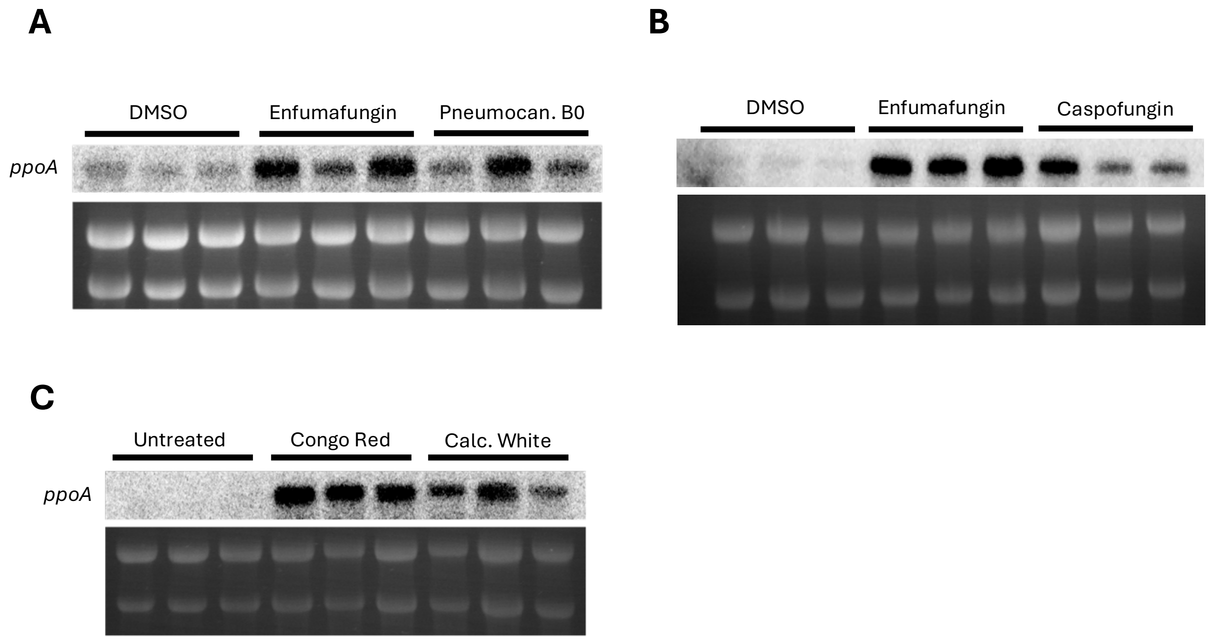

Figure S4. Cell wall stress induces *ppoA* expression. (A) Northern blot analysis of *ppoA* expression in WT Af293 under treatment with vehicle, 1 µg/mL enfumafungin, or 1 µg/mL Pneumocandin B0. **(B)** Northern blot analysis of *ppoA* expression in WT CEA10 under treatment with vehicle, 2 µg/mL enfumafungin, or 2 µg/mL Caspofungin. **(C)** Northern blot analysis of *ppoA* expression in WT Af293 under treatment with vehicle, 50 µg/mL Congo red, 50 µg/mL calcofluor white.

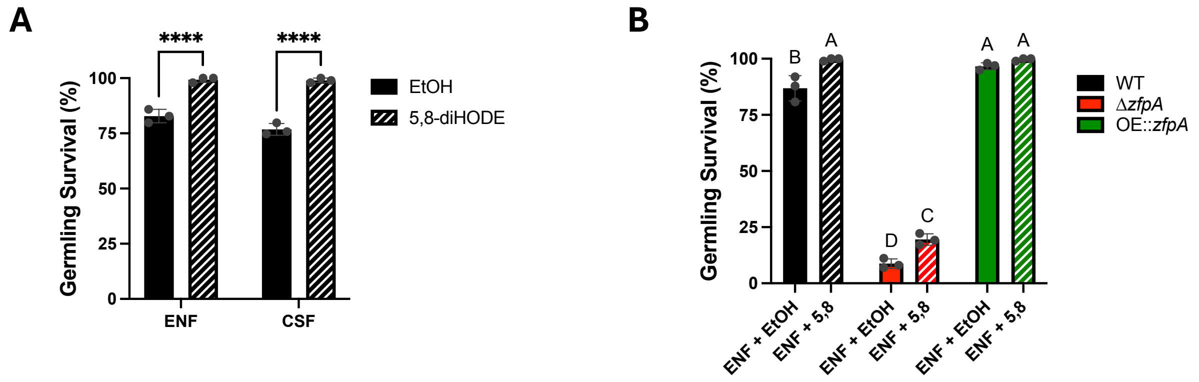

Figure S5. 5,8-diHODE protects against fungicidal tip lysis by enfumafungin in *A. fumigatus* CEA10. (A) Percent survival of *A. fumigatus* CEA10 germlings treated with 2 µg/mL caspofungin or enfumafungin and 1 µg/mL 5,8-diHODE or EtOH vehicle after 16 hours in GMM + 0.01% YE. '***' denotes p<0.001 by Fisher's LSD test. **(B)** Percent survival of *A. fumigatus* CEA10 germlings treated with 2 µg/mL enfumafungin and 1 µg/mL 5,8-diHODE or EtOH vehicle after 16 hours in GMM + 0.01% YE. Conditions with p values less than 0.05 calculated by two-way ANOVA with Tukey's multiple comparisons are indicated by distinct letters.

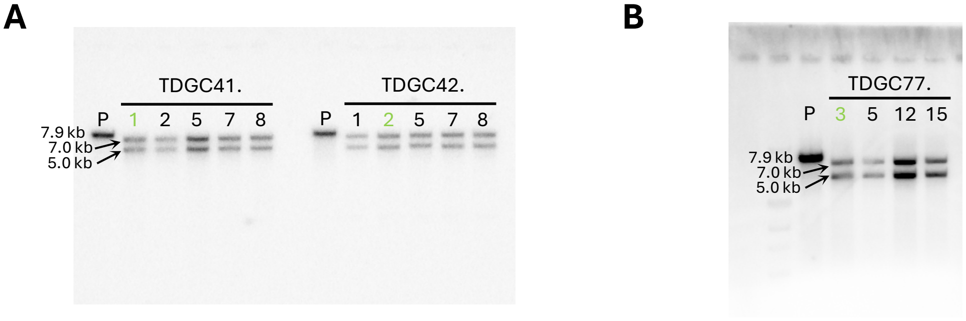

Figure S6. Southern blot confirmation of TetOff::*fksA* strains. (A) Southern blot of AlwNI digested genomic DNA of parental strains TFYL81.5 and TJW214.2 and their respective TetOff::*fksA* mutants. **(B)** Southern blot of AlwNI digested genomic DNA of parental strain TJW213.1 and resulting TetOff::*fksA* mutants.

A

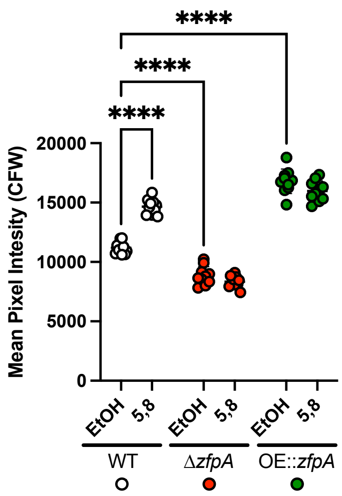

Figure S7. ZfpA is required for increased hyphal chitin in response to 5,8-diHODE treatment. Mean CFW intensity per pixel of ten hyphae grown for 12 hours in GMM + 0.01% YE with 1 μg/mL 5,8-diHODE or 1% EtOH vehicle before staining and epifluorescent imaging. '**' denotes p<0.01 and '***' denotes p<0.0001 determined by Browne-Forsythe and Welch ANOVA with Dunnett's T3 multiple comparisons tests.

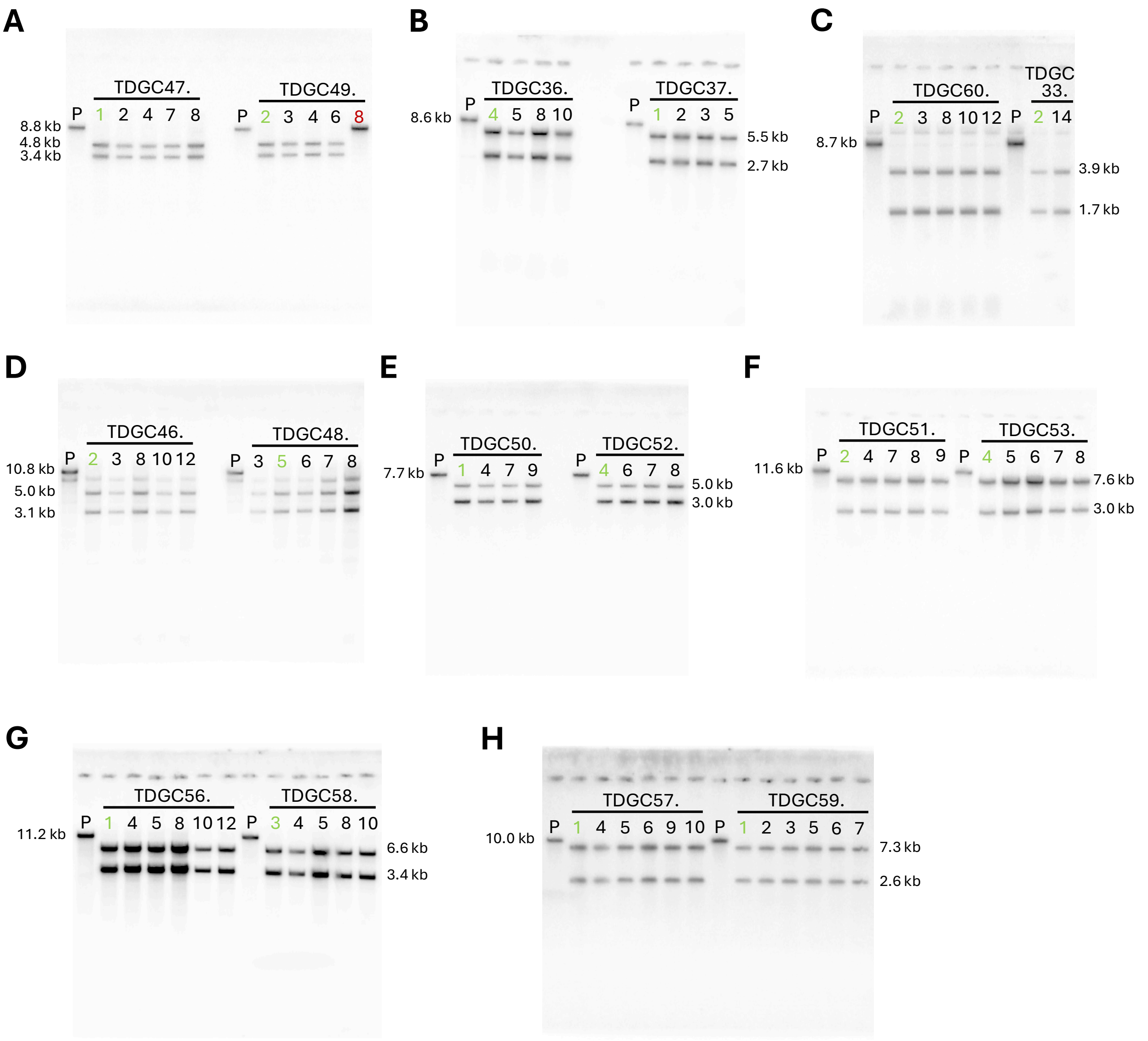

Figure S8. Southern blot confirmation of chitin synthase deletion strains. (A) Southern blot of SapI digested genomic DNA of parental strains TDGC19.1 and TDGC12.2 and their respective Δ*chsA* mutants. **(B)** Southern blot of FspI digested genomic DNA of parental strains TDGC19.1 and TDGC12.2 and their respective Δ*chsG* mutants. **(C)** Southern blot of SnaBI digested genomic DNA of parental strains TDGC19.1 and TDGC12.2 and their respective Δ*csmA* mutants. **(D)** Southern blot of FspI digested genomic DNA of parental

strains TDGC19.1 and TDGC12.2 and their respective Δ*csmB* mutants. **(E)** Southern blot of MfeI digested genomic DNA of parental strains TDGC19.1 and TDGC12.2 and their respective Δ*chsD* mutants. **(F)** Southern blot of SphI-HF digested genomic DNA of parental strains TDGC19.1 and TDGC12.2 and their respective Δ*chsF* mutants. **(G)** Southern blot of MscI digested genomic DNA of parental strains TDGC19.1 and TDGC12.2 and their respective Δ*chsB* mutants. **(H)** Southern blot of MfeI digested genomic DNA of parental strains TDGC19.1 and TDGC12.2 and their respective Δ*chsC* mutants.

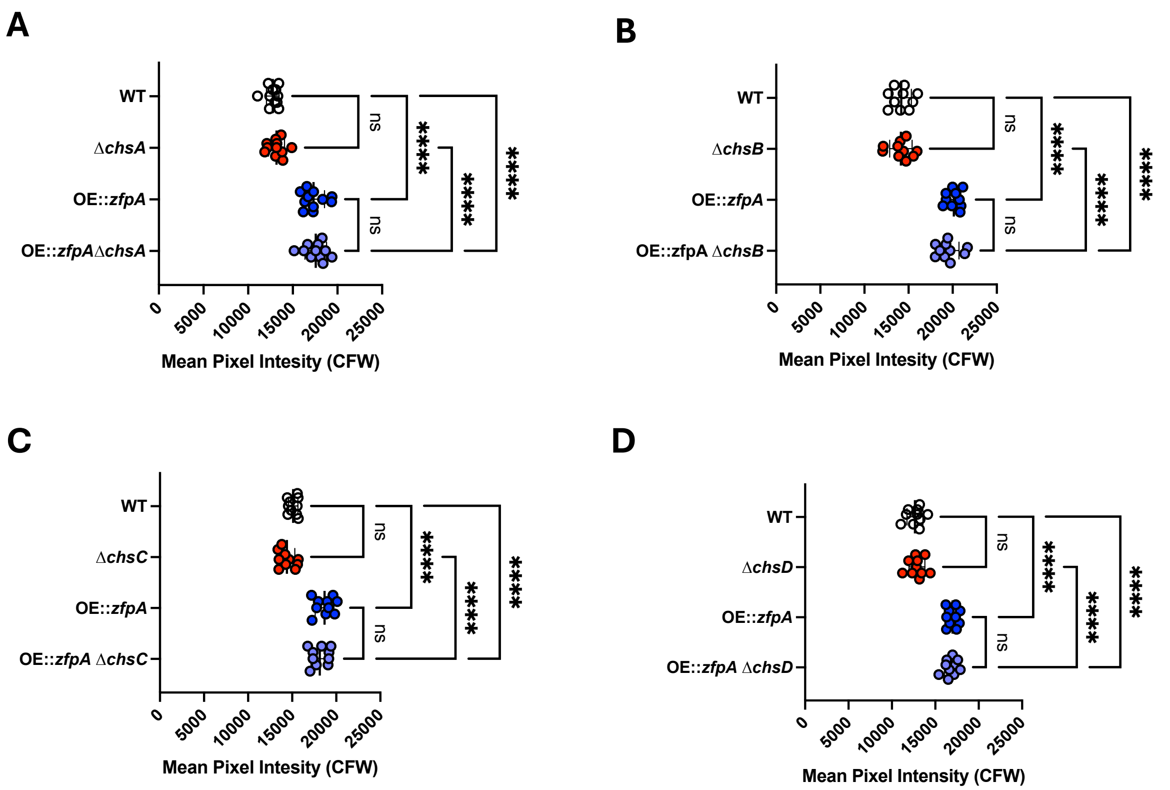

Figure S9. Four chitin synthases are dispensable for normal chitin levels in *A. fumigatus* hyphae. (**A-D)** Mean CFW intensity per pixel of ten hyphae grown for 12 hours in GMM + 0.01% YE before staining and epifluorescent imaging. '****' denotes p<0.0001 determined by Browne-Forsythe and Welch ANOVA with Dunnett's T3 multiple comparisons tests.

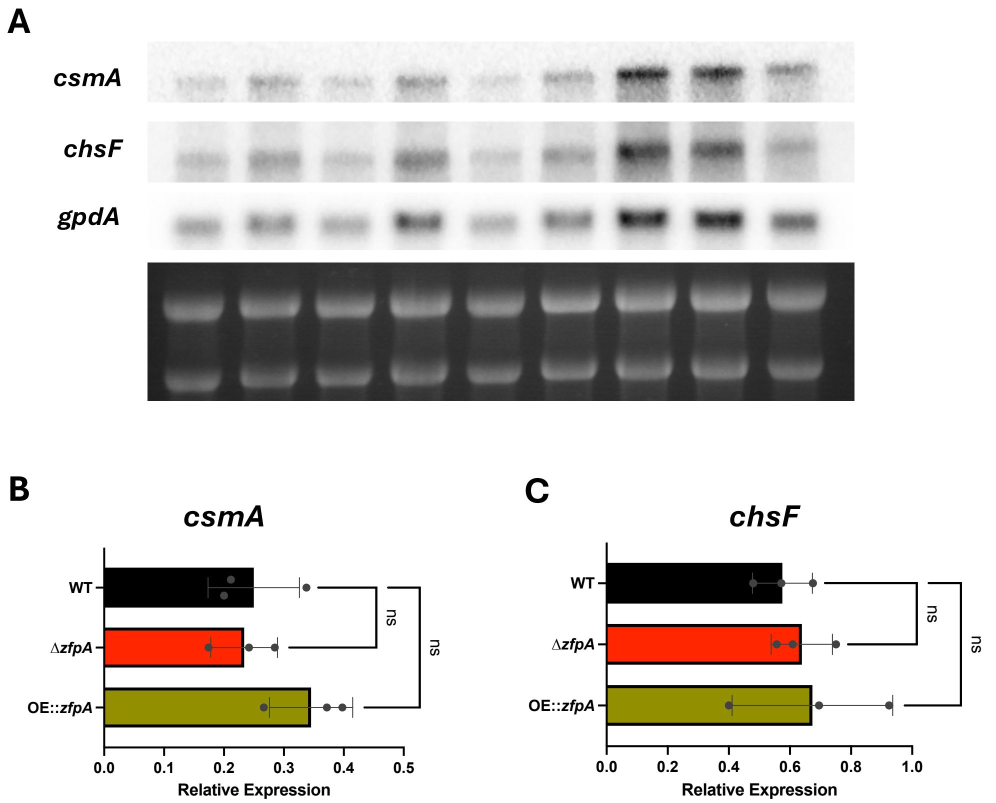

Figure S10. Expression of *csmA* and *chsF* are not altered in ZfpA mutants. (A) Northern blot analysis of *csmA, chsF, and gdpA* expression in WT Af293 after 24 hours in GMM at 37˚C and 250 RPM. **(B)** Mean transcript signal of *csmA* relative to *gpdA* measured using ImageJ. **(C)** Mean transcript signal of *chsF* relative to *gpdA* measured using ImageJ.

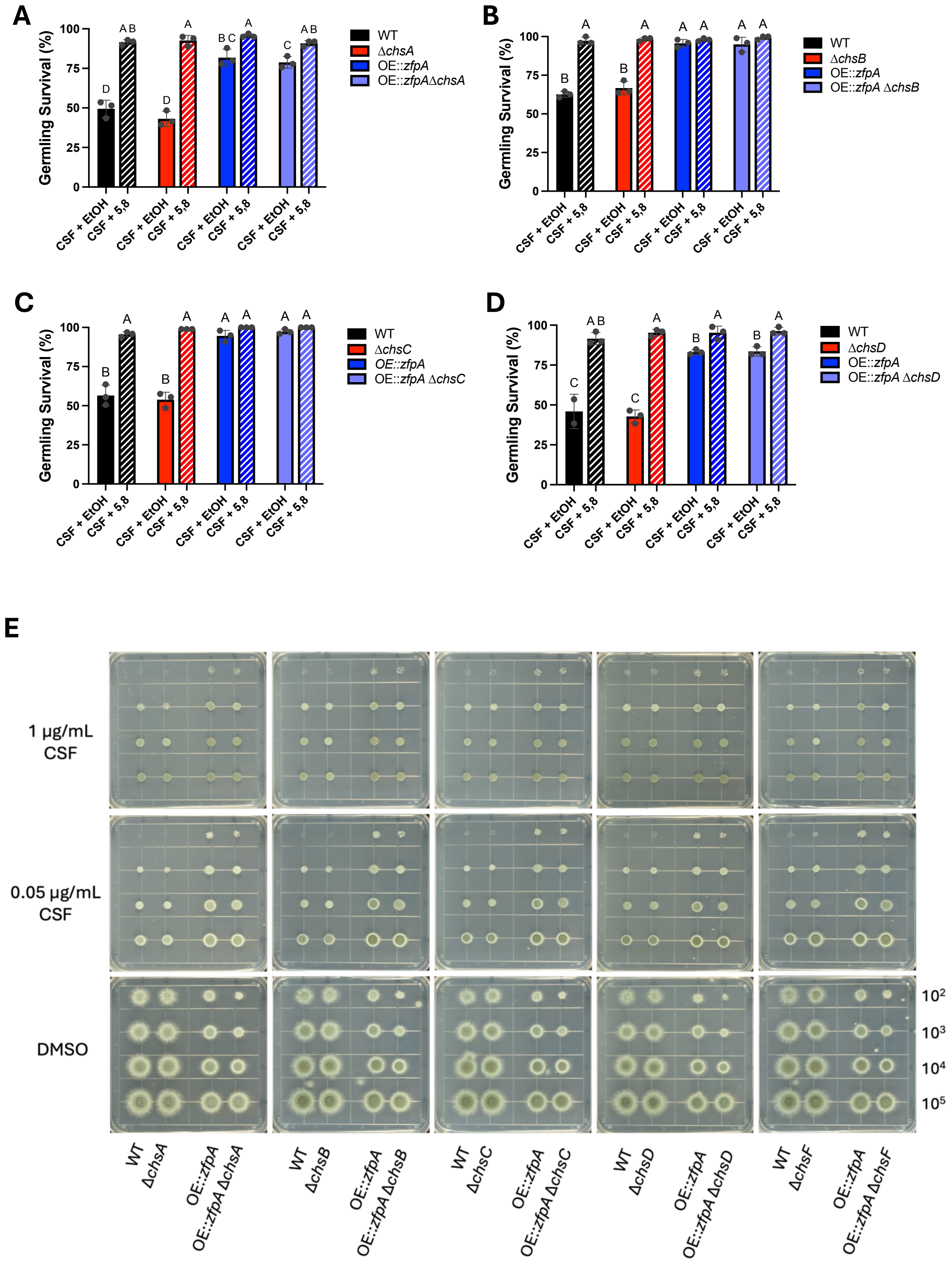

A
B
C
D
E
Germling Survival (%)
CSF + EtOH
CSF + 5,8
WT
ΔchsA
OE::zfpA
OE::zfpAΔchsA
ΔchsB
OE::zfpA ΔchsB
ΔchsC
OE::zfpA ΔchsC
ΔchsD
OE::zfpA ΔchsD
1 μg/mL CSF
0.05 μg/mL CSF
DMSO
10^2
10^3
10^4
10^5
ΔchsF
OE::zfpA ΔchsF

Figure S11. Loss of ChsA, ChsB, ChsC, or ChsD alone does not impact susceptibility to caspofungin. **(A-D)** Percent survival of *A. fumigatus* Af293 germlings treated with 2 μg/mL caspofungin and 1 μg/mL 5,8-diHODE or EtOH vehicle after 16 hours in GMM + 0.01% YE. Conditions with p values less than 0.05 calculated by two-way ANOVA with Tukey's multiple comparisons are indicated by distinct letters. **(E)** *A. fumigatus* Af293 conidia spotted on GMM with according concentrations of caspofungin after incubation at 37 ˚C for 48 hours.

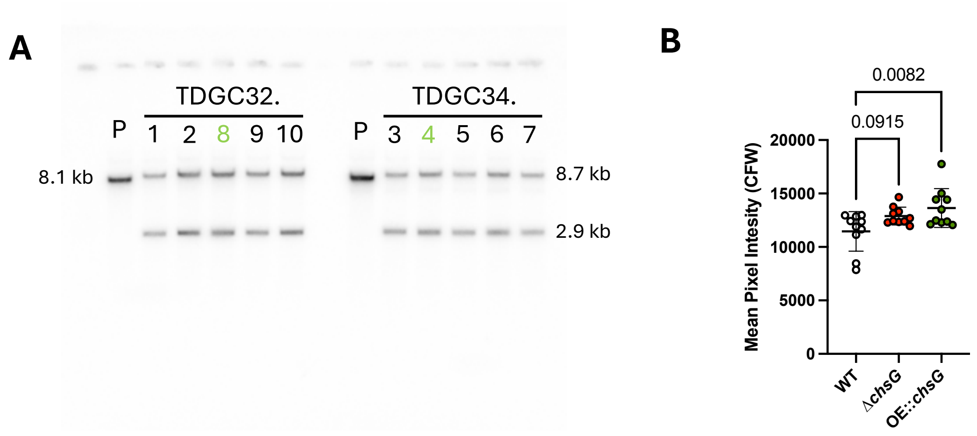

Figure S12. Confirmation of OE::*chsG* mutant strains. (A) Southern blot of ApaI digested genomic DNA of parental strains TFYL45.1 and TFYL80.1 and their respective OE::*chsG* mutants. **(B)** Mean CFW intensity per pixel of ten hyphae grown for 12 hours in GMM + 0.01% YE before staining and epifluorescent imaging. One-way ANOVA with Tukey's multiple comparisons used to calculate p-values shown.

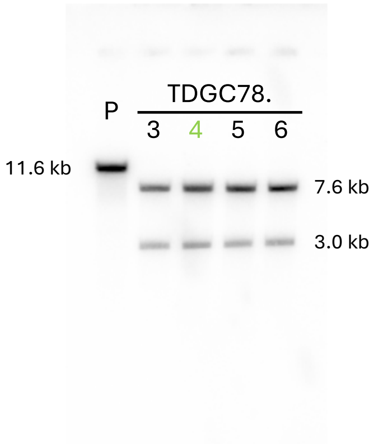

Figure S13. Southern blot confirmation of OE::*chsG* Δ*chsF* mutant strains. Southern blot of SphI-HF digested genomic DNA of parental strain TDGC32.8 and resulting Δ*chsF* mutants.

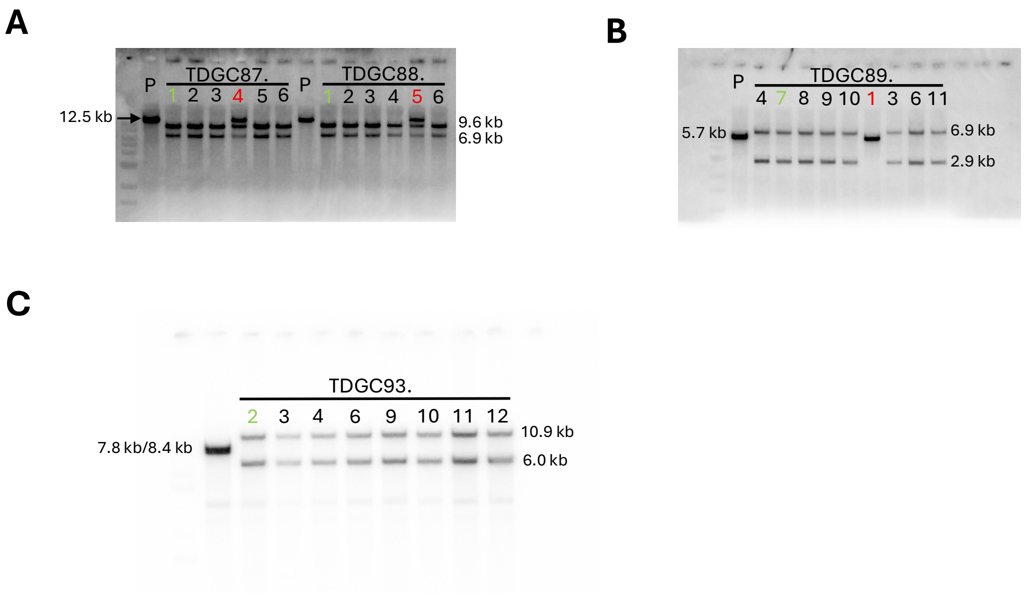

Figure S14. Southern blot confirmation of OE::*chsF and zfpA chsG* double mutant strains. (A) Southern blot of SnaBI digested genomic DNA of parental strains TDGC19.1 and TDGC11.3 and resulting OE::*chsF* mutants. **(B)** Southern blot of EagI-HF digested genomic DNA of parental strain TDGC32.8 and resulting OE::*zfpA* mutants. **(C)** Southern blot of EcoRI-HF digested genomic DNA of parental strain TDGC11.3 and resulting Δ*chsG* mutants.

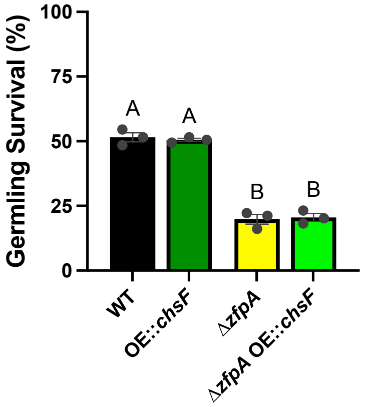

Figure S15. Overexpression of *chsF* does not impact fungicidal tip lysis by caspofungin. Percent survival of *A. fumigatus* Af293 germlings treated with 2 μg/mL caspofungin and 1 μg/mL 5,8-diHODE or EtOH vehicle after 16 hours in GMM + 0.01% YE. Conditions with p values less than 0.05 calculated by two-way ANOVA with Tukey's multiple comparisons are indicated by distinct letters.

Table S1. Table of *A. fumigatus* strains used in this study.

Strain	Background	Genotype	Source
TFYL81.5	Af293	Δ*akuA*	(1)
TJW213.1	Af293	Δ*akuA*; Δ*zfpA*	(2)
TJW214.2	Af293	Δ*akuA*; OE::*zfpA*	(2)
TFYL45.1	Af293	Δ*akuA*; *pyrG-*; *argB-*	(1)
TDGC9.1	Af293	Δ*akuA*; *pyrG-*; Δ*ppoA*	This study
TDGC19.1	Af293	Δ*akuA*; *argB-*	This study
TDGC23.1	Af293	Δ*akuA*; Δ*ppoA*	This study
TDGC24.14	Af293	Δ*akuA*; OE::*ppoA*	This study
WT Af293	Af293	wild type	Clinical isolate
TDWC1.13	Af293	Δ*ppoA*	(3)
TJMP143.28	Af293	OE::*ppoA*	(4)
TDGC11.3	Af293	Δ*akuA*; *argB-*; Δ*zfpA*	This study
TDGC12.2	Af293	Δ*akuA*; *argB-*; OE::*zfpA*	This study
TDGC16.13	Af293	Δ*akuA*; Δ*zfpA*; OE::*ppoA*	This study
TDGC17.1	Af293	Δ*akuA*; OE::*zfpA*; Δ*ppoA*	This study
WT CEA10	CEA10	wild type	Clinical Isolate
TDGC1.2	CEA10	Δ*akuB*; OE::mCherry	(2)
TJW215.1	CEA10	Δ*akuB*; OE::mCherry; Δ*zfpA*	(2)
TJW216.1	CEA10	Δ*akuB*; OE::mCherry; OE::*zfpA*	(2)
TDGC41.1	Af293	Δ*akuA*; TetOff::*fksA*	This study
TDGC42.2	Af293	Δ*akuA*; OE::*zfpA*; TetOff::*fksA*	This study
TDGC77.3	Af293	Δ*akuA*; Δ*zfpA*; TetOff::*fksA*	This study
TDGC47.1	Af293	Δ*akuA*; Δ*chsA*	This study
TDGC49.3	Af293	Δ*akuA*; OE::*zfpA*; Δ*chsA*	This study
TDGC56.1	Af293	Δ*akuA*; Δ*chsB*	This study
TDGC58.3	Af293	Δ*akuA*; OE::*zfpA*; Δ*chsB*	This study
TDGC57.1	Af293	Δ*akuA*; Δ*chsC*	This study
TDCG59.1	Af293	Δ*akuA*; OE::*zfpA*; Δ*chsC*	This study
TDGC50.1	Af293	Δ*akuA*; Δ*chsD*	This study
TDGC52.4	Af293	Δ*akuA*; OE::*zfpA*; Δ*chsD*	This study
TDGC51.2	Af293	Δ*akuA*; Δ*chsF*	This study
TDGC53.4	Af293	Δ*akuA*; OE::*zfpA*; Δ*chsF*	This study
TDGC36.4	Af293	Δ*akuA*; Δ*chsG*	This study
TDGC37.1	Af293	Δ*akuA*; OE::*zfpA*; Δ*chsG*	This study
TDGC60.2	Af293	Δ*akuA*; Δ*csmA*	This study
TDGC33.2	Af293	Δ*akuA*; OE::*zfpA*; Δ*csmA*	This study
TDGC46.1	Af293	Δ*akuA*; Δ*csmB*	This study

TDGC48.5	Af293	Δ*akuA*; OE::*zfpA*; Δ*csmB*	This study
TDGC32.8	Af293	Δ*akuA*; *argB*-; OE::*chsG*	This study
TDGC34.4	Af293	Δ*akuA*; OE::*chsG*	This study
TDGC78.4	Af293	Δ*akuA*; OE::*chsG;* Δ*chsF*	This study
TDGC87.1	Af293	Δ*akuA*; OE::*chsF*	This study
TDGC88.1	Af293	Δ*akuA*; Δ*zfpA*; OE::*chsF*	This study
TDGC89.7	Af293	Δ*akuA*; OE::*zfpA;* OE::*chsG*	This study
TDGC93.2	Af293	Δ*akuA*; Δ*zfpA*; Δ*chGF*	This study

Table S2. Table of oligonucleotides used in this study.

**Primer Name**	**Sequence**
zfpA_1kb_5'flankF	TTATCGTCATCTACCACCCCGG
zfpA_1kb_3'flankR	TCCTTGTCGGATTTAGGAGCGC
zfpA_pJMP10.2overhang_5'flankR	gtacatcatcccataatacttcaagctGCAGACGTCCTAAGCTCGATAG
zfpA_1kb3'ofATG	TCATCAAAGTCTGCGACGCTCG
zfpA_pJMP10.2overhang_ATGF2	ctaccccgcttgagcagacatcaccATGCAGAGCCCAGGAGAACATTCC
ppoA_A.f.argBoverhang_5'flankR	gaaaatttgtcttggatgcagaccgcgttcGGCGGATATAGCTTGCAGAAC
A.f.argBF	gaacgcggtctgcatccaag
ppoA_A.f.argBoverhang_3'flankF	gatcaaatggatgtatgggtctctccttcTCGAGGCTGTGGGTCAAATTGG
A.f.argBR	gaaggagagacccatacatcc
ppoA_pJMP10.2overhang_5'flankR	gtacatcatcccataatacttcaagctGGCGGATATAGCTTGCAGAAC
pJMP10.2F	agcttgaagtattatgggatgatg
ppoA_1kb3'ofATGR	GATCAAGACTACGCCAACGTCC
A.n.gpdA(p)R_DGC	ggtgatgtctgctcaagcggg
ppoA_pJMP10.2overhang_ORFF2	gctaccccgcttgagcagacatcaccATGTCTGAGAAGCAAACCGGTTC
fksA_1kb5'flankF	AACGCCTGCATCATGTTCAACG
fksA_pSK606overhangR	gatgcaagaggccatctaggccatcaGCTGGAATGAAACTGTTGACAGC
pSK606_TetOff(p)F	tgatggcctagatggcctcttg
pSK606_TetOff(p)R	ggtgatgtctgctcaagcgg
fksA_pSK606overhangF	ctaccccgcttgagcagacatcaccATGTCGGGATATCAACAAGGGGG
fksA_1kbR	GGCTTCCAAGGTTTCCTCAACG
csmA_5'flankF	GTCCCGAGGAATCTCCACATCC
csmA_5'flank_overhangR	gaaaatttgtcttggatgcagaccgcgttcTATGTGGATGTTGTCCAGTCCCG
csmA_3'flank_overhangF	gatcaaatggatgtatgggtctctccttcTCTTTGCATCGCCTCATCCACTG
csmA_3'flankR	TGGAAAGATGACATCGTCCGCC
chsG_5'flankF	TCATCGAATCGTTACCCTGCCG
chsG_5'flank_pJMP9overhangR	gtcctctcgggccatctgttcgtataagctGGTGTCGATGCTTTGTCAAGCTG

chsG_pJMP9overhangF	gctaccccgcttgagcagacatcaccATGGCCTACCAAGGCTCTGGTTC
chsG_1kb3'ofATGR	CTTGGTAGACACCGATTGTCGC
chsG_5'flank_overhangR	gaaaatttgtcttggatgcagaccgcgttcGGTGTCGATGCTTTGTCAAGCTG
chsG_3'flank_overhangF	gatcaaatggatgtatgggtctctccttcAGGGGCGTTCTTCGTAATGAGAG
chsG_3'flankR	TGCTCCACTCCCAAGCATCACC
csmB_5'flankF	ATATCAGAAAGGGGAGCCAGGG
csmB_5'flank_overhangR	gaaaatttgtcttggatgcagaccgcgttcGATGACTTAGTTGACCGTATGACGGTAG
csmB_3'flank_overhangF	gatcaaatggatgtatgggtctctccttcCGCCAGATTGACCAGGAGTTGG
csmB_3'flankR	AAGAATCACCACCACCTCAGCC
chsA_1kb5'flankF	GGGAATTTTCCCGAATCTCAAGCG
chsA_5'flank_overhangR	gaaaatttgtcttggatgcagaccgcgttcTGTGAGAGTAGAGATCTTCGGATATTGATC
chsA_3'flank_overhangF	gatcaaatggatgtatgggtctctccttcGCTAATGCTTCGCACAGTTCCG
chsA_1kb3'flankR	GTCATTAACCGTCCCATTGGCG
chsD_1kb5'flankF	TAGGGGAAGAGTATGCAGCAGC
chsD_5'flank_overhangR	gaaaatttgtcttggatgcagaccgcgttcCGCTGGATGCTGTATGTACGG
chsD_3'flank_overhangF	gatcaaatggatgtatgggtctctccttcGGTACTTGCACACGTCGTAAGG
chsD_1kb3'flankR	GCTTGGCATTATGTCAGCAGCC
chsF_5'flank_overhangR	gaaaatttgtcttggatgcagaccgcgttcGACGCTTCTATGGATTCAGAAAGACATTG
chsF_3'flank_overhangF	gatcaaatggatgtatgggtctctccttcGGTATCCGGCACAGTATGAGCC
chsF_1kb3'flankR	ATGCCTCGTTCTGGTTAGTGGC
chsF_1kb3'flankR2	GTGGCGTCTTGATGAGAGAGG
chsB_1kb5'flankF	AATACGTCCACGGATCTCGACG
chsB_5'flank_overhangR	gaaaatttgtcttggatgcagaccgcgttcGGTAGATGGGATCGGATGACTGC
chsB_3'flank_overhangF	gatcaaatggatgtatgggtctctccttcTGATGTCTGGTGACTGTATTCTAGAAAGG
chsB_1kb3'flankR	CGGCCTAAAAGTCGTAGACTGG
chsC_1kb5'flankF	TTCTCCCTCGATCTCATCCACC
chsC_5'flank_overhangR	gaaaatttgtcttggatgcagaccgcgttcTTTGGCAGTGAGCTCTTGAGATTGG

chsC_3'flank_overhangF	gatcaaatggatgtatgggtctctccttcGATAGTTTACTGACCGTGAGACAGAACAC
chsC_1kb3'flankR	AGCGGATGAGCACTTTGTCACC
chsF_1kb5'flankF2	gtaagttgaccctgagcggacc
chsF_5'flank_pJMP10overhangR	gtacatcatcccataatacttcaagctgacgcttctatggattcagaaagacattgtc
chsF_ATG_pJMP10overhangF	gctaccccgcttgagcagacatcaccatgtctttgccccagaggcc
chsF_1.2kbORFR	aaagccgtacaggggaagtacc
chsF_1kb5'flankF3	ctagttcccctgttctggtgtcc
chsF_1kbORFR	ctgctggaagaagaagcttccg
csmA_NorthernF	TCTCTTCGACACCCTGACAACC
csmA_NorthernR	CACTGAAACCTCTTCCCGTTCC
chsF_NorthernF	TGGACGAAGAGAGACTCGAACC
chsF_NorthernR	AGTACACATCACCTGAGCTCCG
A.fu.gpdA_NorthernF	CTCAAATACGACACCACCCACG
A.fu.gpdA_NorthernR	GCTTGATCTCGTCGTAGGAAGC
ppoA_NorthernF	ACAGGACCTCATCAGGACGTTC
ppoA_NorthernR	AAGTTGGTGCAGAGTCCACTGC
zfpA_NorthernF	GATCTATGACCGGAGGTCATCC
zfpA_NorthernR	TTCGTACCATCACCAGCTCCTC
fksA_NorthernF	TCAAAGGTTCTCGCCACAACCG
fksA_NorthernR	TCTCCAACAGCTCGCAGTGTCC

Supplementary References

1. Throckmorton K, Lim FY, Kontoyiannis DP, Zheng W, Keller NP. 2016. Redundant synthesis of a conidial polyketide by two distinct secondary metabolite clusters in Aspergillus fumigatus. Environ Microbiol 18:246–259.

2. Schoen TJ, Calise DG, Bok JW, Giese MA, Nwagwu CD, Zarnowski R, Andes D, Huttenlocher A, Keller NP. 2023. Aspergillus fumigatus transcription factor ZfpA regulates hyphal development and alters susceptibility to antifungals and neutrophil killing during infection. PLOS Pathog 19:e1011152.

3. Dagenais TRT, Chung D, Giles SS, Hull CM, Andes D, Keller NP. 2008. Defects in Conidiophore Development and Conidium-Macrophage Interactions in a Dioxygenase Mutant of Aspergillus fumigatus. Infect Immun 76:3214–3220.

4. Vargas-Muñiz JM, Renshaw H, Waitt G, Soderblom EJ, Moseley MA, Palmer JM, Juvvadi PR, Keller NP, Steinbach WJ. 2017. Caspofungin exposure alters the core septin AspB interactome of Aspergillus fumigatus. Biochem Biophys Res Commun 485:221–226.
